# The Perceptual and Social Components of Metacognition

**DOI:** 10.1037/xge0000180

**Published:** 2016-06-16

**Authors:** Niccolo Pescetelli, Geraint Rees, Bahador Bahrami

**Affiliations:** 1 Department of Experimental Psychology, University of Oxford; 2 Wellcome Trust Centre for Neuroimaging, Institute of Neurology and UCL Institute of Cognitive Neuroscience, University College London; 3 UCL Institute of Cognitive Neuroscience, University College London and The Interacting Minds Centre, Aarhus University

**Keywords:** collective decision making, metacognition, social interaction

## Abstract

When deciding whether or not to bring an umbrella to work, your confidence will be influenced by the sky outside the window (direct evidence) as well as by, for example, whether or not people walking in the street have *their own* umbrella (indirect or contingent evidence). These 2 distinct aspects of decision confidence have not yet been assessed independently within the same framework. Here we study the relative contributions of stimulus-specific and social-contingent information on confidence formation. Dyads of participants made visual perceptual decisions, first individually and then together by sharing their wagers in their decisions. We independently manipulated the sensory evidence and the social consensus available to participants and found that both type of evidence contributed to wagers. Consistent with previous work, the amount people were prepared to wager covaried with the strength of sensory evidence. However, social agreements and disagreement affected wagers in opposite directions and asymmetrically. These different contributions of sensory and social evidence to wager were linearly additive. Moreover, average metacognitive sensitivity—namely the association between wagers and accuracy—between interacting dyad members positively correlated with dyadic performance and dyadic benefit above average individual performance. Our results provide a general framework that accounts for how both social context and direct sensory evidence contribute to decision confidence.

Traditionally, psychology has treated confidence as a subjective, private element of our cognition in the study of choices (Gigerenzer, Hoffrage, & Kleinbölting, 1991; Peirce & Jastrow, 1884; Vickers, 1979). However, confidence is also an essential component of our social life. We recognize confidence in others and value it. We gain or lose confidence by interacting with others. These observations suggest that our sense of confidence is not constructed exclusively from internal states but is also sensitive to social context. Moreover, our subjective sense of confidence—stated verbally or otherwise—also contributes to important social functions such as joint decision making (Bahrami et al., 2010, 2012b; Frith, 2012) and advice taking (Mannes, Soll, & Larrick, 2014) by allowing us to share information about our uncertainty in state(s) of the world around us. In this paper, we investigate these bidirectional impacts of decision confidence and social interaction on one another.

## Perceptual and Social Sources of Confidence

We feel more confident of our choices when they are based on convincing evidence in comparison with when we have to depend on ambiguous information. Numerous works that studied choice confidence in the context of perceptual (Fleming & Lau, 2014; Peirce & Jastrow, 1884) and value-based (De Martino, Fleming, Garrett, & Dolan, 2013; Lebreton, Abitbol, Daunizeau, & Pessiglione, 2015) decision making conceived of decision evidence entirely as information directly pertinent to the immediate choice. A number of elegant computational models have been developed that relate various characteristics of such information, such as signal strength (Kepecs & Mainen, 2012; Ko & Lau, 2012; Pouget, Drugowitsch, & Kepecs, 2016), noise distribution (Budescu, Erev, & Wallsten, 1997), and effector uncertainty (Fleming et al., 2015; Ma & Jazayeri, 2014) to decision confidence (Smith & Vickers, 1988).

But the quality of immediate evidence is not our only source of confidence. We recruit a host of contextual evidence when judging the probability that we have made a correct decision. For example, longer deliberation time reduces confidence even when the quality of evidence is kept constant (Kiani, Corthell, & Shadlen, 2014). Even irrelevant but corollary external information consistent with our choice also increases our confidence. For example, knowing that Parma (but not Venice) has a football club in the Serie A (the Italian national football league) increases people’s confidence in choosing Parma over Venice as the city with larger population (Gigerenzer et al., 1991).

Perhaps the most common example of such confidence boost from ancillary information happens when we realize that others agree with us. In these cases the cue to higher probability of accurate judgment lies in our assumptions about what we believe the others’ agreeing opinions indicate. Statistically, coinciding independent samples (e.g., others’ opinions) decrease our uncertainty about the statistical properties of the phenomenon under investigation. Decreased uncertainty, in turn, can contribute to increased confidence (see next section for further unpacking of this concept).

In addition, social consensus has enormous heuristic value beyond higher accuracy. When in agreement with others, we share responsibility for the choices we make (Harvey & Fischer, 1997) which, in turn, may help us justify our choices and even reduce error costs such as regrets (Nicolle, Bach, Frith, & Dolan, 2011). Moreover, confirmation from others could relieve us of the need to gather extra information through direct experience drastically reducing the costs of decision making. Seeking consensus could also help us learn from social signals in the absence of actual veridical feedback about the accuracy of our choices (Bahrami et al., 2012a).

In summary, both perceptual and social information can change our uncertainty about the states of the external world. Thus, circumstantial social information (e.g., confirmation vs. opposition from others) and directly relevant evidence (e.g., sensory stimulus strength in a perceptual choice) should both contribute to subjective confidence. However, their relative contributions to decision confidence have not been directly compared. Earlier theoretical and empirical works on forecast aggregation (Clemen, 1989; Morris, 1974) have proposed numerous possible schemes for how advice from multiple opinions (i.e., social information) should be aggregated. As if coming from a parallel reality, a rich body of research in system neuroscience on optimal cue combination has offered very similar solutions for how neuronal populations that code different modalities of sensory information should combine their information in multisensory perception. Applied to the context of our study, the question critical to both of these approaches is whether the two sources of information, that is, perceptual and social information are combined linearly to give rise to collective choice and confidence or not. Here, we empirically and directly tested this hypothesis. We asked how much the confirmation of another person increases our confidence in comparison with the increase in confidence attributable to sensory stimulus strength that raises our performance from chance to a prespecified threshold level.

## Individual Differences in Metacognition and Collective Decision Making

In numerous perceptual as well as cognitive decisions as widely divergent as sports refereeing and medical diagnosis, the accuracy achieved by integrating different opinions can exceed the accuracy of each individual opinion, a phenomenon referred to as the “two-heads-better-than-one” effect (Koriat, 2012) or the “wisdom of the crowd” (WOC) (Lorenz, Rauhut, Schweitzer, & Helbing, 2011; Mannes et al., 2014). Early empirical records of this phenomenon date back to the beginning of the last century (Galton, 1907) and numerous theoretical attempts have been made to understand its basis (Bovens & Hartmann, 2004; Condorcet, 1785; Nitzan & Paroush, 1985). The intuition behind the earlier accounts was that any observation is a mixture of information combining the state of the environment (signal) with random noise (error). Assuming that observers are independent in their judgments and not consistently biased toward a preferred belief/decision, pooling observations from different observers together should average out the uncorrelated noise and thus enhance the signal. This notion of the “wisdom of the crowd” is inspired by the concept of repeated measurements in statistics (Armstrong, 2001; Surowiecki, 2004). The same holds true even *within* one observer: better estimates are obtained when the same person gets a chance to combine information over repeated observations (Green & Swets, 1966) or repeated judgments (Rauhut & Lorenz, 2011; Vul & Pashler, 2008). However, some have contended that in many such real-world interactive decisions, agents go beyond simply aggregating their independent samples and also communicate some measure of uncertainty about their observation (Bahrami et al., 2010; Brennan & Enns, 2015). The mental processes involved in estimating the uncertainty in our choices are classified under the more general umbrella-term metacognition (Flavell, 1976).

A distinction has been made between implicit metacognition, defined as those automatic processes of uncertainty monitoring (Bach & Dolan, 2012) and explicit metacognition, defined as a conscious and effortful process that may be a distinctively human ability evolved for social coordination and cooperative behavior (Frith, 2012). This latter view holds that explicit metacognition provides humans with the unique ability of sharing and discussing their own beliefs, perceptions, and intentions, leading to a shared view of the world where fruitful group interactions are facilitated (Friston & Frith, 2015). Indeed, people vary greatly in their capacity to explicitly estimate the uncertainty in their choices (Fleming, Weil, Nagy, Dolan, & Rees, 2010). Moreover these interindividual differences are stable across visual perceptual tasks (Song et al., 2011) but vary across cognitive domains such as perception and memory (Baird, Smallwood, Gorgolewski, & Margulies, 2013).

Many recent studies of metacognition have employed signal detection theory and analysis of behavior in the so-called “type II” decisions (Galvin, Podd, Drga, & Whitmore, 2003; Macmillan & Creelman, 2005) where agents comment on their (often) earlier “type I” decisions. First-order choices (or “type I”) are decisions about characteristics of a physical stimulus (e.g., presence/absence of a signal among noise or categorization of some sensory feature). Second-order (or “type II”) choices are decisions about “type I” decisions that, among other things, may indicate the agent’s level of uncertainty in the accuracy of their Type I decision. For example, confidence ratings (Peirce & Jastrow, 1884), perceptual awareness scale (Overgaard & Sandberg, 2012), and postdecision wagering (Persaud, McLeod, & Cowey, 2007) are forms of type II decisions. The term *metacognitive sensitivity* has been used to refer to the covariation between reported uncertainty and Type I choice accuracy. For example, for an observer with high metacognitive sensitivity, a decision made with high confidence is more likely to be correct than another decision made with low confidence. Several measures have been developed in the literature to characterize such metacognitive sensitivity. Some of them, for example, meta-*d*′, make specific assumptions about the underlying process generating the confidence judgments while others, like the type II A_ROC_, do not (for a detailed description of metacognitive metrics see Fleming & Lau, 2014). Sensitivity of first and second-order decisions are often correlated (Koriat, 2012), meaning that measurement of the sensitivity of the two types of decision can be confounded by each other. However, new empirical methods have been devised to segregate the two (Fleming & Lau, 2014; Song et al., 2011) and measure them independently.

These metacognitive measures of uncertainty have recently been introduced to models of collaborative decision making (Bahrami et al., 2010; Migdal, Rączaszek-Leonardi, Denkiewicz, & Plewczynski, 2012; Sorkin, Hays, & West, 2001). This new approach followed from recent observations that collective benefits of cooperation can exceed what is expected from the purely statistical advantage of vote aggregation (Bahrami et al., 2010; Allison A. Brennan & Enns, 2015). Inspired by the computational principles of optimal cue integration (Knill & Pouget, 2004), Bahrami and colleagues (2010) proposed a Weighted Confidence Sharing (WCS) model for joint decision making. The model posited that, to arrive at a joint decision, interacting agents shared their Type I decisions weighted by their type II decisions which, in this case was their respective confidences. The dyad would then compare these confidence-weighted decisions that support opposite choice alternatives and opt for the choice supported by the higher confidence. This conceptually simple model correctly predicted that joint perceptual decision making would go beyond vote counting but fall short of idealistic Bayesian cue combination which had previously been demonstrated in multisensory perception (Ernst & Banks, 2002).

Even though the WCS model employed the concept of sharing confidence, its predictions for dyadic sensitivity only incorporated each individual’s Type I sensitivity. This was because WCS made the simplifying assumption that participants had a good grasp of their internal uncertainty and could accurately communicate it via confidence sharing (Bahrami et al., 2010). In other words, WCS assumed that interacting individuals’ metacognitive sensitivities are both *good* and *similar* to each other. Since then, empirical evidence for interindividual differences in metacognitive sensitivity (Baird et al., 2013; Fleming et al., 2010; Song et al., 2011) has demonstrated that this assumption was too optimistic. Here we address the question arising from this demonstration: whether, and to what extent, collective decision making depends on interacting individuals’ metacognitive sensitivity. Importantly, to isolate the pure role of metacognitive sensitivity, we were mindful of the frequently observed close association between Type I and type II sensitivity (Barrett, Dienes, & Seth, 2013; Green & Swets, 1966; Kunimoto, Miller, & Pashler, 2001; Maniscalco & Lau, 2012) in our experimental design. We employed a novel, interactive adaptive staircase design to dissociate metacognitive sensitivity from first order sensitivity.

## What Combination Rule Best Captured Confidence Aggregation?

Moreover, as we noted above, the WCS model only predicted the sensitivity of the Type I joint decision making and whether jointly made Type I choices would lead to benefit or loss. The model’s description of the dyadic decision process is abstract and does not provide any clues about psychological mechanisms involved in the confidence of the joint decisions. Critically, it remains agnostic about how interaction and individual confidence sharing may shape the uncertainty associated with the joint decision itself. For example, would the average of individual confidences give a good approximation of the joint confidence? Would it matter for the dyadic confidence if individuals agreed or disagreed with one another? These issues relate directly to the previous section on perceptual and social sources of confidence. To address this question, here we provide a detailed description of the dynamics of dyadic interaction using a novel visualization method. A 2-dimensional Opinion Space is constructed in which each participant’s individual Type I and II decisions are portrayed by a spatial representation along one of the two axes. Locations in this 2-D space correspond to all possible interactive situations. The outcome of the interaction, that is, dyadic Type I and II decisions, are then represented as vectors originating from each location (i.e., interactive situation). Visualization of the vector trajectories on this space helps us understand the dynamics of dyadic interactions.

## Method

### Participants

All participants (*n* = 32; all male; mean age = 24; *SD* = 7) were recruited using the UCL Division of Psychology and Language Sciences’ database of registered volunteers. The choice of recruiting only male participants was motivated by evidence suggesting task-irrelevant sex-stereotypical behavior in mixed-sex dyads and represent common practice in this literature (Buchan, Croson, & Solnick, 2008; Diaconescu et al., 2014; Mahmoodi et al., 2015). Participants came from diverse educational backgrounds and different ethnicities; all of them lived in the U.K. at the time of the study. Participants were paid 7.5£/hour plus possible extra money in case of good performance. Members of each dyad knew each other. The study received ethical approval from the local ethics committee, and written informed consent was obtained from all participants.

### Display Parameters and Response Mode

**Figure 1 fig1:**
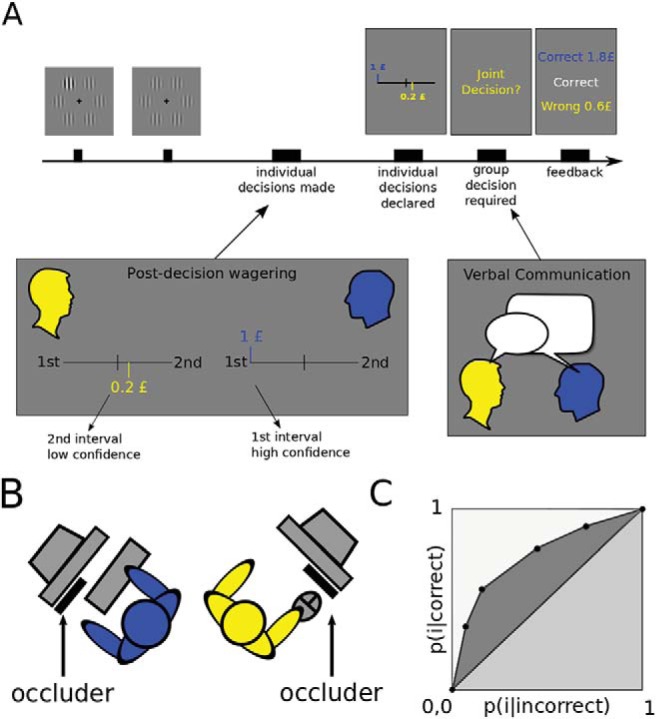
Experimental paradigm. (A) After stimuli were presented on each trial, participants were asked to respond individually through postdecision wagering (PDW) and were not allowed to talk (*Postdecision wagering* panel). Each participant could wager up to one pound on one of two possible intervals. Then, personal decisions were declared and a joint decision was required. Participants could wager together up to one pound on the group’s choice and were now allowed to verbally communicate (*Verbal communication* panel). Finally, feedback on performance and relative earnings were given. (B) Experimental set up: one participant used keyboard response mode and the other mouse response mode. They swapped position and device half way through the experiment. (C) Typical ROC curve constructed from 5-points confidence scale (fictional data). *x* axis: probability of expressing confidence *i* after incorrect decisions. *y* axis: probability of expressing confidence *i* after correct decisions. The area under the curve (A_ROC_ = dark gray + light gray area) represents metacognitive sensitivity. See the online article for the color version of this figure.

The experiment was implemented in MATLAB version 7.6.0.324 (R2008a) (http://www.mathworks.co.uk/) using the Cogentv.1.29 toolbox (http://www.vislab.ucl.ac.uk/cogent.php). Participants sat at right angles to each other, each facing their own LCD Dell monitor (diagonal length = 50 cm, resolution = 800 × 600; Figure 1B). The two monitors were connected to the same Dell Precision 390 (Intel core2 Extreme processor) computer using an output splitter that provided both monitors with the same outputs. Viewing distance was ∼59 cm.[Fig-anchor fig1]

Within each session of the experiment, one participant responded using the keyboard, the other using the mouse. Both participants used their right hand to respond. Each participant in a dyad viewed only half of the screen, with the other half occluded by a piece of thick black cardboard (Figure 1B). The participant using the keyboard viewed the right half of the display; the participant using the mouse viewed the left half of the other display. Halfway through the experiment (i.e., after 128 trials), participants swapped their positions and response devices.

### Stimuli

Six vertically oriented Gabor patches (Figure 1A) (spatial frequency = 1.5 cycles deg^−1^, contrast = .2) were presented for 85 ms equally spaced around an imaginary circle (radius: 8°), followed by a blank display lasting 1000 ms and then another set of gratings for 85 ms (Figure 1A). An oddball of higher contrast, the target, was constructed by adding an additional contrast computed by a modified 2-down-1-up staircase function (Levitt, 1971; Song et al., 2011) to one of the gratings in one of the intervals. The exact locations of the 6 gratings presented in each interval were jittered between 0 and pi/10 arcdegrees on every trial to avoid retinal adaptation. The onset of each interval was jittered between 0 and 500 ms on every trial.

### Task and Staircase

We used a 2-alternative forced-choice (2-AFC) design: participants had to indicate the interval in which the target grating was displayed. Metacognitive sensitivity was probed while maintaining constant accuracy. Metacognitive sensitivity and accuracy are closely correlated and if we allow both to vary independently, it is impossible to disentangle the contribution of metacognitive sensitivity to collective decision making from that of accuracy (Koriat, 2012). To maintain constant accuracy levels, we used a 2-down-1-up staircase procedure to modify the contrast of the target relative to the other nontarget gratings which converged at 70.7% accuracy (Fleming, Huijgen, & Dolan, 2012; Fleming et al., 2010; Levitt, 1971; Song et al., 2011). An important modification was introduced to the algorithm that enhanced the stability of the staircase (Treutwein, 1995) by adaptively reducing the step size at every reversal of direction of decision accuracy (i.e., from error to correct and vice versa) until the minimum step size of 1% luminance contrast was reached. This adaptive adjustment of step size helps stabilize the staircase: as the staircase goes on, step size is adaptively reduced to achieve appropriate precision for threshold measurement, tuning the staircase to each participant’s sensitivity landscape.

### Experimental Conditions and Procedure

**Figure 2 fig2:**
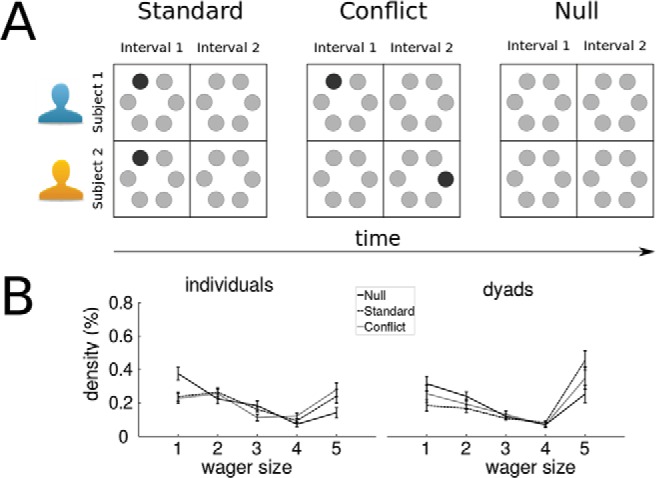
(A) The three conditions employed in the experiment. In Standard trials the oddball target appeared in the same location and interval for the two participants. In conflict trials the oddball appeared in random locations but opposite intervals for the two participants. In Null trials the oddball was indistinguishable from the distractors. (B) Average distributions of individual and dyadic wager size across the three conditions. Wager size is defined as absolute wager rank and ranges from 1 (minimum wager level) and 5 (maximum wager level). Error bars represent *SEM*. See the online article for the color version of this figure.

Three conditions were employed and randomly shuffled across the experiment (Figure 2A). In the Standard condition (Figure 2A, left panel), the oddball appeared in the same location and interval on each trial for both participants. Target contrast was independently computed for each participant by the staircase procedure on the basis of the participant’s previous history of correct/incorrect responses in the preceding Standard trials. In this condition, participants received trial-by-trial feedback about accuracy. There were 156 Standard trials. In the Conflict trials, the oddball appeared in different locations and intervals on each trial for the two subjects (Figure 2A, middle panel). In the Null trials, there was no target at all: the target additional contrast was zero (Figure 2A, right panel). In the Conflict and Null trials, feedback was not provided. There were 50 Conflict and 50 Null trials. Note that (a) target stimulus contrast was at threshold and (b) agreement and disagreement were likely to happen in any of the 3 conditions and (c) whether or not feedback was going to be provided would only be revealed at the end of each trial after all individual and joint decisions had been made. These factors ensured that the participants remained naïve about the conditions throughout the experiment.[Fig-anchor fig2]

The experiment started with a practice block of 16 trials with a fixed target contrast arbitrarily set (well above threshold) at 20%. The main experiment consisted of 2 runs of 8 blocks with 16 trials each. In every trial, participants first made a private decision about the interval the target appeared in. Type II responses were elicited through postdecision wagering (PDW) (Persaud et al., 2007): participants could wager up to one pound in steps of £0.20 on one out of two possible intervals depending on their level of confidence (Figure 1A, “Post-decision wagering” box, color code represents participant). Using forced choice design meant that wagering zero was not allowed. During this individual wagering, participants could not see their partner’s choice and were instructed not to communicate any information about their response. After each participant placed his wager, the computer displayed both participants’ decisions and wagers on the screens (Figure 1A) and a joint decision was prompted. At this stage participants made a joint decision and placed a joint wager of up to one pound on their dyadic choice (not shown in the figure). We defined dyadic deliberation time as the interval from the presentation of the prompt asking for joint decision until the joint decision was declared. The joint decision was communicated to the computer (“confirmed”) by the participant using the keyboard on odd trials and by the participant using the mouse on even trials. Color codes were used to denote the participant using the keyboard (in blue) and mouse (in yellow). Joint decision was elicited by the same color code to indicate which participant was assigned to input the joint decision (Figure 1A). During the collective part, participants could openly discuss their choice and wager (“Verbal Communication” box). Joint wagers were elicited on every trial. In Standard trials, once the joint response was announced, the computer displayed feedback indicating each participants’ earnings and the accuracy of the individual and joint decisions (Figure 1A). Earnings were calculated by adding up the outcomes of the individual and joint wagers. In case of correct decisions the amount of money was positive while in case of incorrect decisions it was negative. For example, suppose that a participant placed 20p on the second interval for the individual decision and agreed to place 80p on first interval for the group decision. Now, if the correct choice turned out to be the first interval, then the participant’s total earnings would be (−20)+80 = 60p. In Conflict and Null trials, feedback was not given and the message ‘Go for the next trial’ appeared instead. The choice of providing feedback only on Standard trials was motivated by the fact that in Null trials accuracy, and hence feedback, could not be defined. Similarly, on Conflict trials, dyadic accuracy could not be defined and providing conflicting feedback for individual choices would give away the experimental manipulation. At the end of the experiment, five trials were randomly selected from each run and participants received 50% of their earnings from these trials. The experimenter was always present in the room to make sure instructions were followed.

**Figure 3 fig3:**
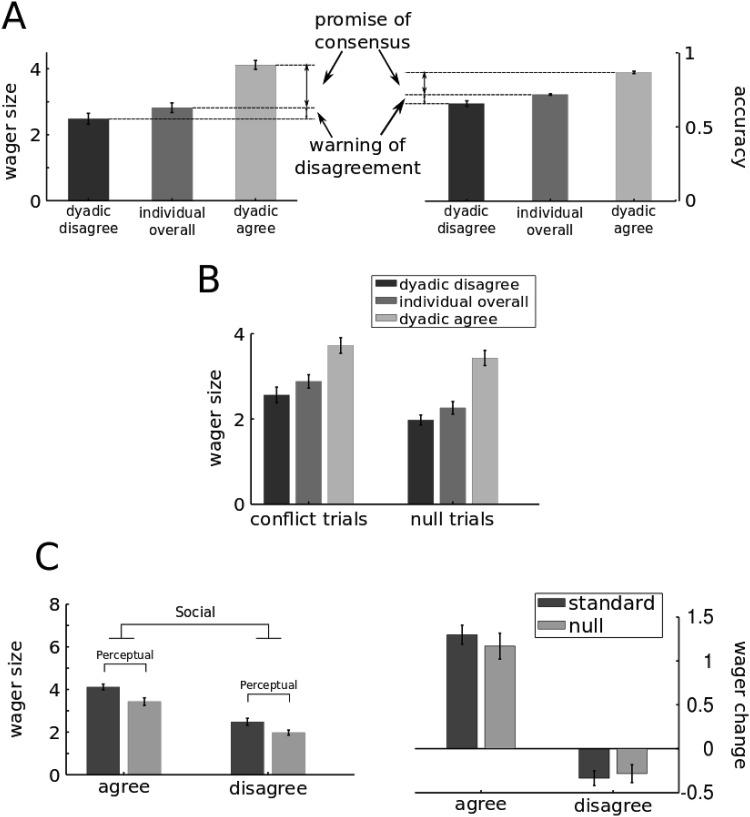
In all panels, “Individual overall” refers to measures taken during the first part of each trial, when individuals made private decisions. The term overall refers to the fact that trials were not split according to social consensus. “Dyadic dis/agree” refers to measures taken in the second part of each trial by both individuals jointly. These measures are split and presented according to consensus. (A) Relationship between changes in wager size and accuracy at the individual (middle bars) and dyadic level (left and right bars) in Standard trials. After interaction, wagers increase or decrease according to social consensus. The magnitude of the change reflects the magnitude of change in the expected correct response rates. (B) Same data as in panel A left, but for Conflict and Null trials. Average wager size across Conflict and Null conditions, different decision types (individual vs. dyadic) and divided by consensus. As in panel A, individual wagers are represented by the middle bar, whereas dyadic wagers are represented by the left and right bars and divided by consensus. (C) Social versus perceptual effect on dyadic wager size (left) and wager change from baseline (right).

Wagers (ranging from 0.2£ to 1£) were analyzed and plotted as wager rank (from 1 to 5) (Figure 2B and Figure 3) to simplify the notation and computation of type II ROC curves. This linear transformation does not affect our data analysis. We refer to absolute wager rank as *wager size* and to signed wager rank simply as *signed wager*, where the sign represents the interval chosen.

### ROC Curves

We assessed participants’ metacognitive sensitivity using the type II receiver operating characteristic (ROC) curve (Macmillan & Creelman, 2005; Song et al., 2011). Although other measures of metacognitive abilities, such as meta *d*′ are sometimes preferred, we opted for the parameter-free type II A_ROC_ because it makes fewer assumptions regarding the underlying generative process for confidence (Fleming & Lau, 2014) and it has been widely used in the literature (Baird et al., 2013; Fleming et al., 2010; Song et al., 2011).

Following signal detection theory, we defined the area under the ROC curve (A_ROC_) as our objective measure of metacognitive sensitivity. The 5-point wager-scale was used as an indirect measure of confidence (Seth, 2008). Specifically, for every wager level *i,* probabilities p(i|correct) and p(i|incorrect) were first calculated (Kornbrot, 2006; Song et al., 2011), transformed into cumulative probabilities, and plotted against each other anchored at [0,0] and [1,1] (Figure 1C). We calculated the area under the curve by following the method provided by Fleming and Lau (2014) which corrects for Type I confounds. All the analyses were performed using MATLAB (Mathworks).

### Aggregate and Trial-Level Models

We tested our hypotheses both at the participant level with ANOVAs (with participant as the unit of analysis) as well as at the trial-level using multilevel models. The use of a multilevel modeling in the trial-level analysis was motivated by the fact that observations of participants within dyads are more likely to be clustered together than observations across dyads. In addition, this approach has several other advantages over ANOVA and traditional multiple linear regressions. (Clark, 1973; Forster & Masson, 2008; Gelman & Hill, 2007). We implemented multilevel models using the MATLAB *fitlme* function (Mathworks) and REML method. In each case, we started by implementing the simplest possible regression model and progressively increased its complexity by adding predictor variables and interaction terms. Within each analysis, models were compared by computing the AIC criterion that estimates whether the improvement of fit is enough to justify the added complexity.

### Wagering in Opinion Space

**Figure 4 fig4:**
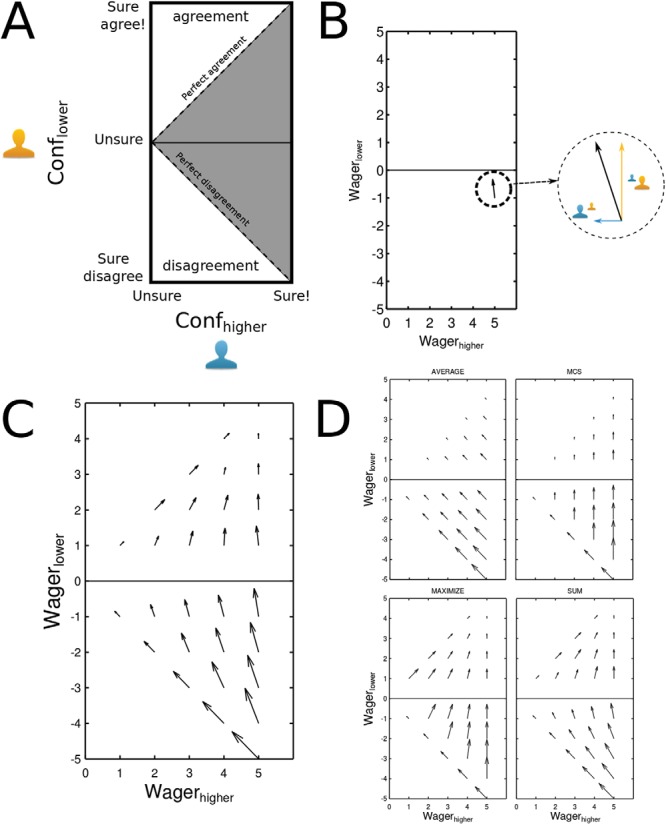
Dyadic Opinion Space. (A) Dynamics of opinions aggregation can be understood by conceiving the dyad as moving along the two-dimensional space whose axes represent each subject’s confidence or postdecisional wagering on any 2AFC task. *x* axis represents wager size of the most confident participant. *y* axis represents wager of the less confident participant relatively to the first participant. Bottom and upper halves represent disagreement and agreement situations respectively. Diagonals represent situations where both subjects placed the same bet on the same (perfect agreement) or opposite intervals (perfect disagreement). The shaded area represents portion of the space where interaction takes place. (B) Each vector‘s components on the grid represent wager change along the scale for each participant. Direction and magnitude represent wager change (Δwager), defined as the signed difference between the average dyadic wager and individual wagers for a particular interactive situation. (C) Empirical vector field averaged across dyads. (D) Vector fields computed on nominal dyads obtained by predetermined algorithms applied to the empirical individual wagers. On each trial and for each dyad a nominal dyad’s response is obtained by computing the wager that the algorithm specifying that nominal dyad would have responded had it been in the situation defined by that trial’s individual private wagers. In particular, bounded *Summing* always sums the two initial individual wagers to obtain the dyadic one. *Maximize* puts the maximum wager on the alternative supported by the most confident participant. *Averaging* always averages the two initial wagers to obtain the dyadic wager. *Maximum Confidence Slating* selects on each trial the wager and choice of the more confident participant and chooses randomly when wagers are equal. Notice the similarity between the bounded *Summing* algorithm and the empirical dyad. See the online article for the color version of this figure.

To better understand the psychological mechanisms of joint decision making, and specifically, to see how interaction and sharing of individual wagers could shape the uncertainty associated with the joint decision, here we introduced a new visualization method. We envisioned the dyadic interaction as movements on a two-dimensional space. Each point on this space corresponds to an interactive situation that the dyad might encounter in a given trial. The *x* coordinate of such point corresponds to the more confident participant’s individual wager on a given trial. The *y* coordinate corresponds to the less confident participant’s choice and wager relative to the first participant: positive (upper half) indicates that the less confident partner’s choice agreed with the more confident partner. Vice versa negative (lower quadrant) indicates disagreement. The triangular area between the diagonals and the *y* axis (Figure 4, shaded area) indicates the space of possible interactive situations.[Fig-anchor fig4]

In any trial, participants may start from a given point on this space (i.e., during the private wagering phase). Via interaction they make a joint decision and wager. This final outcome of the trial can also be represented as a point on this space. Because the dyadic choice and wager are the same for both participants, these points will all line on the agreement diagonal (i.e., 45 degree line in the upper part). Thus, each interaction could be represented by a vector, originating from the coordinates defining private opinions (i.e., choices and wagers) and terminating at some point along the agreement diagonal. We summarize all such interaction vectors corresponding to the same initial point by averaging the coordinates of their termination. The resulting vector (after a linear scaling to avoid clutter) gives an indication of the dyadic strategy. By repeating the same procedure for all possible pairs of private opinions, we chart a vector field that visualizes the dyadic strategy. Our 2-D space consists of a 5x10 “opinion grid” corresponding to the 5 × 10 possible combinations of private opinions (i.e., choices and wagers). Because of the symmetry of our data, trials from the two intervals are collapsed together. To determine the interaction vector for each node of the opinion grid, we took all trials in which private opinion pair corresponded to that node and averaged the differences between the dyadic and private wagers. For example let’s say we want to compute the wager change vector for the node (5, −1) (Figure 4B). We select all trials in which one of the participants reported a wager size of 5 (i.e., maximum wager on either of the intervals) and the other participant disagreed with a wager size of 1 (lowest wager on the other interval). Now we calculate the average dyadic wager on this subset of trials relative to the most confident participant (i.e., positive indicates dyadic wager agrees with most confident wager, negative indicates dyadic wager disagrees with most confident participant) and call this real number *k*. The *x* and *y* components of a wager change vector are defined as: *x*’ = *k* − (5) and *y*’ = *k* − (−1). Linear rescaling was applied so to fit all arrows to the size of the grid according to the quiver MATLAB function.

This simple measure represents both the direction and magnitude of wager change as a consequence of interaction. The main descriptive strength of this visualization is that we can apply the same procedure to nominal dyads that, in each trial, take the same private wagers—namely same *x* and *y*—but follow a specific strategy (e.g., averaging individual wagers) to reach a dyadic choice—namely *k*—and compare the resulting nominal Opinion Spaces to our empirically obtained one. The comparison (see Results) provides an immediate and intuitive understanding of the dyadic strategy employed. We compare the empirical dyads with five different strategies for wager aggregation: (a) Averaging: signed private wagers are averaged together. In case of disagreement ties the minimum wager on a random interval is made; (b) Maximum Confidence Slating: The interval and wager of the more confident person are taken as dyadic interval and wager. In case of disagreement ties, one of the two participants’ intervals is taken randomly; (c) Maximizing (Supplementary material): the interval chosen by the more confident participant is taken as dyadic interval and the maximum wager possible (i.e., 5) is taken as wager size. In case of disagreement ties, one of the two participants’ intervals is taken randomly; (d) Summing: signed wagers are added up together and bounded by the maximum wager available (i.e., 5). In case of disagreement ties the minimum wager on a random interval is made (c) Coin Flip (Supplementary material): one of the two participants’ interval and wager is taken at random as dyadic interval and wager.

### Control Measures

After the experiment each participant was tested with two brief computer-based tasks that assessed individual economic personal traits like risk and loss aversion that could have confounded our PDW measures. Neither our Risk-Aversion nor Loss-Aversion index correlated with any of the variables of interest; both individual and dyadic levels were considered (see Supplementary material for details). Relevant personality traits were also assessed for each participant using two online questionnaires (see Supplementary material for further details and results).

## Results

### Frequency of Agreement in Different Conditions

Manipulation of perceptual evidence affected the frequency of agreements significantly across the three conditions (one-way ANOVA *F*(2, 30) = 50.9, *p* < .001, η_G_^2^ = .64). Agreements were most frequent in the Standard trials (∼60%), significantly more frequent than in Null trials (∼50%), *t*(15) = 4.86, *p* < .001, *d* = 1.69, which in turn contained significantly more agreements than Conflict trials (∼40%), *t*(15) = 4.47, *p* < .001, *d* = 1.44.

### Visual Signal Drives Individual Confidence

At the participant level, mean individual wager size differed across conditions (Standard trials = 2.82, Conflict = 2.88, Null = 2.26, *F*(2, 62) = 77.81, *p* < .01, η_G_^2^ = .09) (Figure 2B left panel, Figure 3A and 3B). Post hoc comparisons showed that individual wager size for Standard and Conflict trials did not differ significantly but were both significantly greater than Null trials (paired *t* test; both *t*(31) > 8.8, both *p* < .001, *d* > 0.7). Figures S3–S8 show the distribution of wager sizes for each participant and dyad across the three conditions. These results serve as reassuring sanity check by confirming that individuals’ confidence behavior did follow and reflect the availability of perceptual information in the Standard and Conflict trials compared with Null trials where no visual signal had been presented to the participants.[Fig-anchor fig3]

### Perceptual and Social Sources of Confidence

To address our first theoretical question and quantify the contribution of social and perceptual information to dyadic uncertainty, we asked how the perceptual manipulation and the emerging consensus influenced dyadic wagers. We will first present the results from multilevel model analysis and report the results both for standardized and unstandardized variables. After reporting each significant effect using the multilevel analysis, we will report the equivalent finding using the more conventional ANOVAs in which participant is the unit of analysis (effect sizes are reported as Generalized Eta Squared [η_G_^2^]; Bakeman, 2005). This slightly redundant approach allowed us to communicate the findings more intuitively and to make sure the results did not arise from some particular artifact of the method being used.

#### Linear mixed effect modeling results

To understand the factors influencing dyadic wagers, we employed a multilevel linear regression with trials as data points; importantly we defined individual trials as grouped within participants themselves grouped within dyads. We tested several models to predict dyadic wager size (DV). The winning model according to the AIC criterion (Akaike, 1974) had five predictors and 10 fixed coefficients (Table S1a). Main fixed-effect predictors were consensus (coded categorically as 0 for disagreement and 1 for agreement), condition (coded categorically as 0 for Null, 1 for Standard, and 2 for Conflict), and absolute individual wager size (assumed to be continuous, ranging from 1 to 5). Their reciprocal interactions were also added to the model as fixed-effects terms. At the dyadic level, a random term was defined only for the intercept. At the subject level random-effects were defined for intercept, for each main predictor and for two interaction terms, namely agreement*condition and agreement*individual wager. The random-effect interaction between individual wager and condition was not included because it did not significantly improve the fit of the model, χ^*2*^(19) = 22.51, *p* > .2. The resulting model was significantly better than a model without random effects and multilevel structure as tested by a Likelihood Ratio Test, χ^*2*^(37) = 2544.5, *p* < .001.

We predicted that both private wagers and social information (e.g., consensus) should affect dyadic wagers. Indeed beta coefficients (see SM Table S1a for complete table) showed that dyadic wager was positively predicted by both individual wager size (β = 0.40, *SE* = 0.04, β_std_ = 0.36, *SE*_std_ = 0.03, *p* < .001) and by agreement compared to disagreement (β = 1.27, *SE* = 0.18, β_std_ = 1, *SE*_std_ = 0.06, *p* < .001). Moreover both Standard (β = 0.58, *SE* = 0.10, β_std_ = 0.21, *SE*_std_ = 0.05, *p* < .001) and Conflict (β = 0.60, *SE* = 0.11, β_std_ = 0.18, *SE*_std_ = 0.05, *p* < .001) conditions predicted larger dyadic wager size compared to Null condition.

#### ANOVA results

We combined the data from 32 individuals and 16 dyads into a unified analysis by taking the average dyadic wagers input (“confirmed”) by each individual separately and construct a 2-way repeated-measures ANOVA (3 conditions: Standard, Conflict, Null × 3 decision types: individual, dyadic agree, dyadic disagree) with mean absolute wager size as the dependent variable. We found a main effect of condition, *F*(2, 62) = 62.68, *p* < .001, η_G_^2^ = .07, main effect of decision type, *F*(2, 62) = 110.14, *p* < .001, η_G_^2^ = .32, and a significant interaction between the two, *F*(4, 124) = 6.34, *p* < .001, η_G_^2^ = .01 (Figure 3A, left panel and Figure 3B). Planned comparisons confirmed that dyadic wagers were indeed higher for agreement trials compared to disagreement trials, *t*(31) = 14.26, *p* < .001, *d* = 1.69 and to individual private wagers, *t*(31) = 9.94, *p* < .001, *d* = 1.29; dyadic wagers in disagreement in turn were significantly smaller than individual wagers, *t*(31) = −3.5, *p* = .001, *d* = 0.38. Within agreement trials, average dyadic wager size in Standard trials was significantly greater than in Conflict trials, *t*(31) = 4.81, *p* < .01, *d* = 0.38; wager size in Conflict trials was, in turn, significantly greater than in Null trials, *t*(31) = 2.75, *p* < .01, *d* = 0.29. Within disagreement trials on the contrary no difference was found between Standard and Conflict trials but these conditions showed greater wagers than Null trials, *t*(31) > 5.1, *p* < .001, *d* > .55.

### Testing the Predictions of the Optimal Cue Combination Theory

Optimal cue combination (Knill & Pouget, 2004) would predict (see Introduction) that under the Null condition in which the perceptual cues are less reliable or simply nonexisting, dyads should rely more heavily (compared to Standard condition) on social cues such as consensus.

#### Linear mixed effect modeling results

The Standard condition did not interact with consensus (β = −0.01, *SE* = 0.09, β_std_ = −0.006, *SE*_std_ = 0.06, *p* > .9), meaning that the difference in dyadic wager between agreement and disagreement trials was equal in Standard and Null trial. The Conflict condition, on the contrary, interacted negatively with consensus (β = −0.2, *SE* = 0.10, β_std_ = −0.12, *SE*_std_ = 0.06, *p* = .04): compared with Null trials the consensus effect (the difference in dyadic wager between agreement and disagreement trials) was reduced in this condition.

Moreover, trial-by-trial private wager size interacted negatively with both Standard (β = −0.08, *SE* = 0.02, β_std_ = −0.07, *SE*_std_ = 0.02, *p* < .001) and Conflict (β = −0.10, *SE* = 0.02, β_std_ = −0.09, *SE*_std_ = 0.02, *p* < .001) conditions in predicting dyadic wager size. This means that the positive relation between individual wager size and dyadic wager size observed in Null trials was reduced in the other two conditions. In the absence of a perceptual evidence (i.e., a Null trial), dyadic wagers followed the initial individual opinions closely and contrary to predictions of optimal cue combination, social interaction did not add much variance, whereas when the stimulus was presented, social interaction contributed more significantly to dyadic wagers, making it more difficult to predict the dyadic wager size from individual wagers size only. Note that in Figure 3C, social interaction was operationalized by agreement versus disagreement whereas here social interaction is inferred from the trial-by-trial predictive relationship between individual and dyadic wagers. Individual wager size and consensus interacted positively (β = 0.13, *SE* = 0.05, β_std_ = 0.12, *SE*_std_ = 0.05, *p* = .01). Compared with disagreement trials, the regression factor relating individual and dyadic wager sizes became more positive under agreement. This finding is indicative of a change in dyadic wagering strategy that depended on the social situation (i.e., agreement vs. disagreement). We will come back to this point further below (see Opinion Space in empirical and nominal dyads).

#### ANOVA results

To disentangle the role of social information from stimulus strength at the participant level, we studied within-condition wagers across decision types. By comparing agreement and disagreement trials in Standard and Null conditions we were able to disentangle the social and perceptual components of wager change (Figure 3C). In particular, differences in wager size between agreement and disagreement (the social effect) were compared when stimulus was present (Standard) versus when stimulus was absent (Null). A 2-way repeated measures ANOVA (2 consensus levels: agree vs. disagree × 2 stimulus levels: present (Standard trials) vs. absent (Null trials)) showed significant effects both for consensus, *F*(1, 31) = 248.91, *p* < .001, η_G_^2^ = .45, and stimulus factors, *F*(1, 31) = 107.88, *p* < .001, η_G_^2^ = .11, but, critically, no interaction. The same was true when the ANOVA had as dependent variable wager change from baseline (i.e., the respective individual wager corresponding to each dyadic decision type) instead of wager size. The results did not show any interaction between the social and the perceptual factors (*p* > .22; Figure 3C, right panel). Moreover, whereas the consensus effect (Agree vs. Disagree) was maintained, *F*(31) = 248.91, *p* < .001, η_G_^2^ = .60, the effect of stimulus presence (Standard vs. Null) was now absent (*p* > .5) indicating that wager change due to interaction (i.e., difference between the private and dydic wager) was not affected by stimulus presence.

Taken together, the multilevel modeling and ANOVA results showed that social interaction per se did not modulate the uncertainty about stimulus strength, but contributed to dyadic wager by providing some extra piece of independent evidence (i.e., agreement or disagreement). The dyadic wagers reflected both the social and the perceptual evidence additively and linearly. The consensus effect (i.e., the difference between agreement and disagreement trials) was the same for Standard and Null trials. These findings did not seem to confirm the prediction drawn from Optimal Cue Combination.

#### Did dyadic deliberation time impact the joint interaction?

Another question that only the trial-by-trial analysis could address is whether dyadic deliberation time (see Methods) impacted the dyadic wagers. We expanded our model to include a main regressor for dyadic deliberation time (Table S1b). A negative significant effect for deliberation time in predicting the dyadic wager was obtained only from standardized data (β = −0.01, *SE* = 0.007, β_std_ = −0.08, *SE*_std_ = 0.008, *p* < .001). It suggests that lower deliberation times are associated with greater dyadic wagers. The only interaction effect that survived the likelihood ratio test was that deliberation time interacted negatively with individual wager size (β = −0.008, *SE* = 0.002, β_std_ = −0.03, *SE*_std_ = 0.009, *p* < .001). This is plausible because highest dyadic wagers are made when dyad members are confident and they reach a joint decision quickly.

### Wager Changes Reflect Expected Accuracy Rates

As shown in Figure 3, in all conditions consensus increased wager size to a significantly greater extent than disagreement reduced it, *t*(31) > 2.52, *p* < .02, *d* > 0.77. We tested whether this pattern of dyadic wagering parallels a similar statistical regularity in the choice accuracy. If so, then agreements and disagreements should differently predict the success of dyadic perceptual judgments. In Standard trials, we compared dyadic accuracy conditioned on agreement versus disagreement with the overall individual accuracy. This way, we directly tested whether the observed boost in wager size attributable to agreement was indeed coupled with a similar boost in the dyadic accuracy. We restricted our analysis to Standard trials because these are the only trials where dyadic accuracy can be defined meaningfully. A “promise of consensus” measure was defined as the difference between average dyadic wager size (or accuracy) in agreement trials and average individual wager size (or accuracy). Similarly a “warning of disagreement” was defined as the difference between average individual wager size (or accuracy) and the average dyadic wager size (or accuracy) in disagreement trials (Figure 3A). Paralleling the earlier findings on wager size, the promise of consensus for accuracy was significantly greater than the warning of disagreement, *t*(31) = 4.33, *p* < .001, *d* = 1.13 (Figure 3A, right). In addition, the difference between the promise of consensus and the warning of disagreement was calculated for wager and accuracy measures. These two differences were positively correlated across dyads, *r*(30) = .34, *p* = .05, suggesting that wager changes after interactions reflected the expected changes in correct response rate. Importantly, such positive relationship observed between wagers and accuracy was present only after social interaction took place. The same analysis on *private* correct response rates showed that such a close match did not exist at the individual level, *r*(30) = .20, *p* = .25. Here the warning of disagreement was significantly greater than the promise of consensus, *t*(31) = 4.30, *p* < .001, *d* = 0.96. Interaction thus led to a better wager-accuracy recalibration. The same result was shown when specifying the nested structure of our data (subjects within dyads) in a simple multilevel regression with subjects as data points (Table S3). In it we chose as our dependent variable the difference between promise of consensus and warning of disagreement for accuracy (DV) and tested whether one could predict this by observing differences between promise of consensus and warning of disagreement for wagers (IV). Once more trials were grouped within participants who in turn were grouped within dyads. Random intercepts were defined for dyads and for participants. Their reciprocal relation was marginally significant (β = 0.04, *SE* = 0.02, β_std_ = 0.34, *SE*_std_ = 0.17, *p* = .05), thus supporting the results obtained by the simple Pearson’s correlation. Moreover, metacognitive sensitivity computed on dyadic choices and wagers was greater than the less metacognitive participants within each dyad, *t*(15) = 2.62, *p* < .02, *d* = 0.79, but no different from the more metacognitive ones (*p* > .4), suggesting that metacognitive accuracy at the dyadic level did not suffer a collective loss.

### Social Influence Analysis

Because a decision and a wager were elicited both before and after social interaction took place on every trial, we were able to investigate the impact of social interaction on dyadic wager directly by looking at the distance between individual and dyadic wager (Δwager). In particular, we were interested in looking at which factors better predicted the more influential individual within each dyad on a given trial.

On Standard trials, because of the staircase procedure, participants agree correctly on .7 × .7 = 49% of trials and incorrectly on .3 × .3 = 9% of trials. So they should have learnt that when they agree, they should trust their judgment. When they disagree on the contrary, they would be correct only 50% of the time if there were to flip a coin between the two of them. But as it can be seen in Figure 3A, right panel, dyadic choices in disagreement are far better than chance, *t*(31) = 8.32, *p* < .001 rejecting the coin flipping as a strategy. Thus, participants are not just randomly choosing between their two judgments. What cue are they following? At the moment of the dyadic choice, when accuracy has not been yet revealed, only choices, current wager sizes and past outcomes are available. Although past accuracy is equal because of the staircase procedure, participants might have learnt who has collected more money so far, which would correspond closely to their own and their partner’s metacognitive sensitivity (see Metacognition and Collective Decision-making). On the other hand, they may follow a much simpler strategy of favoring the partner with higher wager in that trial. In fact, recent works (Mahmoodi et al., 2015) suggest that even when a conspicuous accuracy gap separates the partners, they still insist on following the simpler strategy of choosing the maximum wager. We thus wanted to see whether individuals’ wager size or their metacognitive sensitivity better predicted the influence they exerted on the final dyadic choice and wager. We reasoned that the smaller the distance between the dyadic wager and the individual wager the higher that individual’s influence on the collective final decision. We defined influence (*I*) by:

I=10−|Δwager| 1

where *Δwager = wager*_*dyad*_ *− wager*_*indiv*_ represents the distance between dyadic and individual wager in a given trial. Given this formulation, *I* = 10 would correspond to maximum influence (the individual completely dominated joint wager); conversely, *I* = 0 would indicate minimum influence that is, the individual’s maximum wager on a choice alternative was completely reversed in the dyadic stage. Notice how this measure is tied to the specific scale used and to the private initial wager. For example minimum influence can be achieved only when starting from a wager size of 5. One could think about more sophisticated indexes that measure influence relatively to the starting point (that thus are independent from scale and initial wager size). The downside of more sophisticated measure is that they are harder to interpret.

A multilevel regression was employed (Table S4a) with dependent variable: influence (*I*), predictors: individual wager size, cumulative earnings, condition, and their reciprocal interactions. Trials were grouped within participants and participants within dyads; random intercepts were defined at both levels. The results showed that the only factor determining influence was wager size (β = 0.26, *SE* = 0.03, β_std_ = 0.18, *SE*_std_ = 0.02, *p* < .001) but not earnings that were negatively related with influence (β = −0.002, *SE* = 0.001, β_std_ = −0.05, *SE*_std_ = 0.02, *p* < .02) (Table S4a). Moreover, the effect changed according to conditions. Compared with Null trials, there was a significant positive interaction between absolute individual wager size and Standard trials (β = 0.20, *SE* = 0.04, β_std_ = 0.14, *SE*_std_ = 0.02, *p* < .001) and a marginal negative interaction with Conflict trials (β = −0.08, *SE* = 0.05, β_std_ = −0.06, *SE*_std_ = 0.03, *p* = .07). This suggests that the positive relation between individual wager size and influence was the strongest in Standard, the weakest in Conflict trials, with Null trials lying in between. These findings show that the more influential partner within a dyad was not necessarily the one who was more metacognitively sensitive (i.e., the one with greater A_ROC_), but the one who, so to speak, shouted louder and wagered higher.

It could be the case however that although individual wager size was immediately available to participants, learning who earned more or who was the more metacognitively sensitive partner might have required more time and sampling. The strength of the trial-by-trial analysis is that we could test this hypothesis by including time as a regressor in our model. We added trial number as an extra predictor and looked at its interaction terms with earnings and individual wager size (Table S4b). No positive interaction was found between earnings and time, failing to support the hypothesis that participant learned about metacognitive sensitivity over time. Instead, the influence of the partner with more earnings (hence more metacognitively sensitive) diminished as a function of time (β = −1.8e-5, *SE* = 8.49e-6, β_std_ = −0.02, *SE*_std_ = 0.01, *p* < .05). If anything, more metacognitive partners lost influence with time.

### Opinion Space in Empirical and Nominal Dyads

To visualize the dynamics of opinions integration we looked at the changes in postdecisional wagering on a 2-dimensional Opinion Space, described in the Methods. The results are shown in Figure 4C (Figure S11 shows the plot per each dyad). Point of strongest agreement, namely (5, 5) works as attraction point of the Opinion Space where vectors seemed to converge to. The magnitude of the wager change was maximal along the disagreement diagonal with vectors pointing centrally. Conversely, the vector magnitudes were smallest along the agreement diagonal with vectors pointing externally. These opposite patterns suggested that the dyadic wagering strategy might have changed depending on social context (agreement or disagreement). Indeed, when we compare the empirical findings (Figure 4D) to nominal dyads following some plausible dyadic decision making strategies such as Maximum Confidence Slating (Koriat, 2012), and Averaging (Clemen & Winkler, 1999)—depicted in the top and middle panel of Figure 4D—neither one captures the variability in the empirical data. When in disagreement participants tended to average their wagers by moving toward each other on the scale. On agreement trials, on the contrary, dyads followed a maximizing strategy as they went for the maximum wager level. However, we found that an even simpler strategy, namely simple bounded Summing of signed wagers (Figure 4D, bottom-right panel) captures the empirical findings with remarkable concordance. According to this strategy, dyads aggregate individual wagers simply by adding private wagers bounded of course by the maximum wager size.

**Figure 5 fig5:**
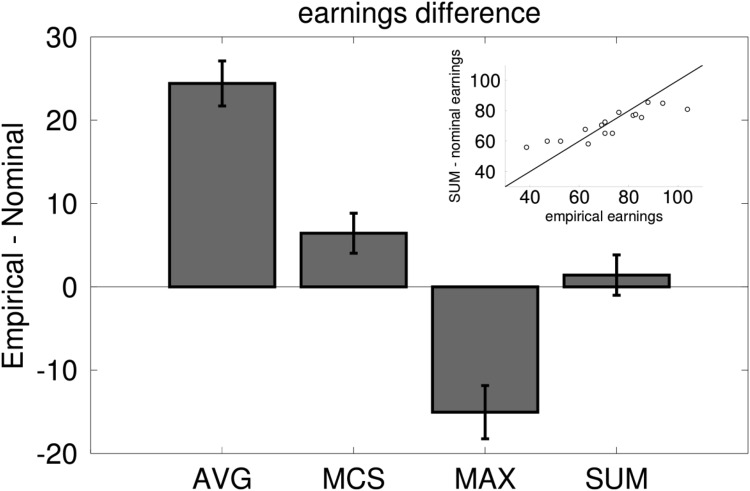
Difference between Empirical and Nominal dyads’ earnings. Positive bars mean that the strategy underperformed empirical dyads and negative bars mean that the strategy outperformed empirical dyads. Inset: Correlation between empirical and nominal earnings as predicted by the SUM strategy. Data points correspond to each dyad. A strong positive correlation, *r*(14) = .88, *p* < .001, demonstrates that the SUM strategy is likely to have been used by the majority of dyads.

To go beyond the qualitative description of the visualization and compare the empirical dyads to the nominal ones arising from each strategy, we compared them on first and second order performance. Specifically we compared the empirical and nominal in terms of proportions of accurate responses and total earnings. Although no difference was found for accuracy (*p* > .9), empirical and nominal dyads faired very differently in terms of earnings for the participants, which directly relates to second-order accuracy (see “Metacognition and Collective Decision-making” below). To compare the similarity of empirical dyads’ strategy with nominal dyads, we computed the difference between empirical earnings and the earnings that participants could have gained had they adopted each nominal strategy (see Figure 5). Positive difference would indicate that dyads performed better than a given strategy would have earned them and vice versa. Across dyads, a one-way ANOVA showed significant differences between the four different strategies, *F*(3, 45) = 75.05, *p* < .001, η_G_^2^ = .63. Planned comparisons showed that both the Averaging and Maximum Confidence Slating strategies significantly underperformed compared to the empirical dyads (both *t*(15) > 4.43, both *p* < .001). On the contrary the wager Maximizing strategy (see Methods) significantly outperformed empirical dyads, *t*(15) = −4.31, *p* < .001, whereas the Summing strategy came closest to the empirical earnings (*p* > .5). This result clearly supports the view that the Summing strategy is the closest description to what we observed empirically. A strong positive correlation, *r*(14) = .88, *p* < .001, between nominal and empirical earnings (Figure 5, inset) suggests that Summing was an adequate descriptor for the majority of dyads and was not an artifact of averaging over dyads.[Fig-anchor fig5]

Importantly, participants did not choose to benefit from the remarkably simple and financially effective strategy of opting for the maximum wager for all dyadic decisions. We will come back to this point in the Discussion.

### Metacognition and Collective Decision Making

**Figure 6 fig6:**
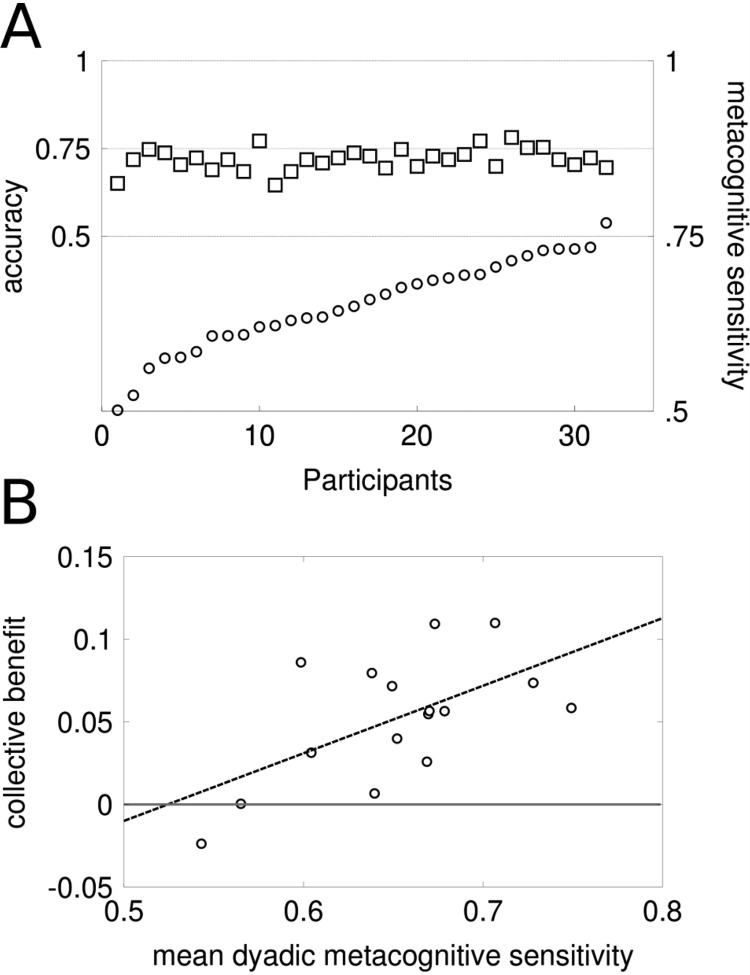
(A) Individual A_ROC_ (circles) and accuracy (squares) values are plotted for each subject. Manipulation of performance with staircase method made different individuals converge around 71% of accuracy. Metacognitive sensitivity was not affected as can be seen by the wide range of A_ROC_ values. The same plot but arranged by dyads is shown in Figure S9A. (B) Correlation between mean dyadic metacognitive sensitivity (computed as A_ROC_) and achieved collective benefit (difference between dyadic accuracy and average participants’ accuracy), *r*(14) = 0.59; *p* = .01. The black solid line indicates the boundary of collective benefit and collective loss. Points above the line indicate dyads reaching collective benefit. Points below the line indicate collective loss.

As expected from the experimental design, performance accuracy converged to 71% (Figure 6A, S9A) and showed very little variance across participants (*M* = 0.72, *SD* = 0.03). Most importantly, accuracy did not show any significant correlation neither with contrast threshold nor A_ROC_ (both *p* > .1; Pearson *r* < .3). Our method was therefore successful at dissociating metacognitive sensitivity from performance accuracy. No matter how well or badly calibrated our participants were, the use of the staircase ensured that all of them experienced an almost identical number of error and correct outcomes. This means that the participants in each dyad could not draw any judgments about one another’s decision reliability by simply counting their errors. In addition to the above, a negative correlation was found between participants’ A_ROC_ and contrast threshold, *r*(30) = −0.38; *p* = .02, as well as a significant positive correlation between participant’s A_ROC_ and total earnings, *r*(30) = .36; *p* = .04. It is important to note that participants were never able to compare their own visual stimulus with that of their partner and were not given any explicit information about each other’s cumulative earnings.[Fig-anchor fig6]

One of our main hypotheses concerned the relation between participants’ metacognitive sensitivity and their success in collective decision making. For each dyad, we calculated the dyadic metacognitive sensitivity by averaging dyad members’ A_ROC_. To assess collective benefit, we calculated the difference between dyadic accuracy in all Standard trials and the average accuracy of individuals working as a dyad. Note that the staircase procedure did not apply to the dyadic decisions and therefore dyadic accuracy was not bound to converge to any predefined level. Dyadic metacognitive sensitivity was significantly correlated with collective benefit (*r*(14) = .59; *p* = .01; Figure 6B, S9B). Dyads formed by individuals who were more able to reliably communicate internal uncertainty were indeed better able to utilize collaboration and enhance dyadic performance.

## Discussion

Numerous previous studies that addressed interactive decision making and opinion aggregation (Bahrami et al., 2010; Kerr & Tindale, 2004; Sorkin et al., 2001) principally focused on the factors that affect collective choice accuracy. The uncertainty and confidence (Pouget et al., 2016) associated with those collective choices has been much less studied. To address this question we tested human dyads making individual and joint perceptual decisions in a visual search for contrast oddball task. Perceptual information (i.e., luminance contrast) was either provided at threshold level titrated for each individual (Standard and Conflict trials) or not at all (Null trials). Social context (agreement vs. disagreement) arose from combinations of individual choices. Confidence judgments (using postdecision wagering) before and after social interactive choice took place was compared under combinations of perceptual and social contexts (see Figures 1 & 2).

We pursued three main theoretical motivations. First, combining the previous works in social psychology of expert forecast aggregation (Clemen, 1989) with the more recent findings in neurobiological basis of optimal cue combination (Trommershäuser, Kording, & Landy, 2011), we asked whether interacting human agents adjust the contribution of perceptual and social information to their joint uncertainty dynamically when making joint decision and confidence. Second, we asked what confidence combination rule could best describe how interacting agents combine their confidences to arrive at joint confidence. The predictions from several plausible theoretical propositions (averaging [Clemen & Winkler, 1999], maximum confidence slating [Bang et al., 2014; Koriat, 2012], maximizing, and bounded summing) were drawn and compared to the data. Finally, we questioned a key assumption of some recent previous works on joint decision making (Bahrami et al., 2010; Koriat, 2012; Sorkin et al., 2001) assuming that interacting agents have similar metacognitive sensitivity and can communicate subjective probabilities equally accurately. In what follows, we unpack how the reported data informs each theoretical issue.

### Testing the Predictions of Forecast Aggregation and Cue Combination Theories

The principal problem addressed in the field of forecast aggregation (Clemen, 1989; Silver, 2012; Tetlock & Gardner, 2015) is to find effective way(s) to combine subjective probability estimates (e.g., 5 year survival rate of a given cancer treatment) from different sources (e.g., two oncologists). Joint perceptual decision making is a natural candidate for solutions proposed by forecast aggregation. Optimal cue integration theory (Knill & Pouget, 2004; Ma, Beck, Latham, & Pouget, 2006; Seilheimer, Rosenberg, & Angelaki, 2014) is the much more recent adaptation of the exact same forecast aggregation problem to system neuroscience. Unsurprisingly, forecast aggregation based on opinion reliability (Morris, 1974) and optimal cue combination (Knill & Pouget, 2004) make similar predictions and prescriptions for how the dyads should combine social and perceptual information. One prediction confirmed by our data was the close correspondence found between changes in wager size and expected accuracy conditioned on consensus (i.e., agreement vs. disagreement). Compared with overall individual accuracy, agreement boosted dyadic accuracy and wager much more than disagreement reduced them. The covariation between confidence and individual accuracy is a well-documented (Fleming & Lau, 2014) but controversial (Krug, 2007; Roediger, Wixted, & Desoto, 2012) phenomenon. Many of these previous works argued for a relationship between private, internal perceptual decision variable(s) and subjective probability of accurate choice (Aitchison, Bang, Bahrami, & Latham, 2015; Meyniel, Schlunegger, & Dehaene, 2015; Pleskac & Busemeyer, 2010). To our knowledge, this is the first report of covariation between confidence and accuracy at joint level. The pattern of results observed here suggested that dyads had a remarkable implicit grasp of the underlying correlation structure between individual choices and their implication for joint accuracy. Dyadic wagers matched the probability of dyadic success. As such, dyadic wagering behavior demonstrated the participants’ deep understanding of the statistics of the social interaction.

Another prediction of forecast aggregation and cue combination theories is that the contribution of each source of information to the joint decision and confidence should depend on the source’s reliability. If perceptual information is weak or nonexisting (e.g., Null trials) then consensus should make a bigger impact on contribution on joint confidence. The prediction drawn from this idea is a statistical interaction in Figure 3C and 3D: the difference between joint confidences under agreement versus disagreement should be larger under Null versus Standard condition. However, the data did not support this prediction. The impacts of perceptual and social factors on wager size were linearly separable. Both the ANOVA and LME analyses showed that the consensus effect—namely the difference between the increase in confidence attributable to agreement and the decrease in confidence attributable to disagreement—has the same magnitude irrespective of the strength of physical evidence provided (i.e., stimulus present in Standard and stimulus absent in Null). The lack of interaction in the ANOVA analysis could not be attributed to averaging over trials since the same result was obtained using trial-by-trial analysis. We will come back to how the observed linear separability could be of help to infer the dyadic strategy for combining individual confidences.

A different counterintuitive prediction of the forecast aggregation and cue combination theories relates to the difference between Conflict and Standard trials conditioned on agreement. The consensus effect (i.e., the difference between joint confidences under agreement vs. disagreement) was significantly smaller for Conflict compared with Null and Standard conditions. Importantly, private wager sizes in Standard and Conflict conditions were indistinguishable (Figure 2B). But upon agreement, dyadic wagers were higher in Standard versus Conflict conditions. This finding is important because the participants did not know about the possibility of conflicting perceptual information. Consequently, they had no reason to entertain the possibility that an agreement may be a “misguided” one arising from one person having made an individual mistake. Nonetheless, and remarkably so, dyadic confidences arising from such misguided agreement in Conflict trials were more modest compared to dyadic confidences arising from true agreements in Standard trials.

This intriguing finding is consistent with forecast aggregation/cue combination if we note that true and misguided agreement trials (in Standard and Conflict conditions, respectively) pooled together different proportions of correct and incorrect individual choices. In a misguided agreement, one of the two agents has made a mistake. Consequently, in Conflict agreement trials, exactly half of the individual decisions were correct. On the contrary, true agreement emerged in about ∼58% of the total number of Standard trials which comprised of ∼49% when both individuals were correct (.7 × .7) in addition to ∼9% when they were both incorrect (.3 × .3). Thus the proportion of correct individual decisions in true agreements was 49/58 = 84%, much higher than 50% observed in Conflict agreement trials. Combining this fact with the much replicated confidence-accuracy correlation follows that the mistaken partner of a misguided agreement should have contributed a lower wager to the joint decision (see Figure 4). This could be attributable to changes of mind (Resulaj, Kiani, Wolpert, & Shadlen, 2009), some postdecisional evidence accumulation process (Pleskac & Busemeyer, 2010; Yeung & Summerfield, 2014), or simply the awareness of weak higher likelihood of error due to unconvincing perceptual evidence. Although changes of mind are often observed under speed pressure, postdecision processes might have contributed to final wagers in Conflict trials here too. Reliability-based forecast aggregation (as well as optimal cue combination) would then require a lower joint confidence under misguided agreement in Conflict condition.

### What Combination Rule Best Captured Confidence Aggregation?

Several previous works have proposed and empirically tested various joint decision rules for how human agents combine choices across individuals (Bahrami et al., 2010; Bang et al., 2014; Koriat, 2012; Migdal et al., 2012; Sorkin et al., 2001). But what combination rule could best describe how interacting agents aggregate confidences? Our experimental paradigm and data allowed us to explicitly write down several distinct and plausible confidence aggregation strategies and apply each one to the data from individuals and draw parameter-free predictions about the dyadic choice and confidence. Some of these plausible strategies were inspired by previous research. We tested averaging (Clemen, 1989), maximum confidence slating (Bang et al., 2014; Koriat, 2012), maximizing, and bounded summing. Interestingly, all of these strategies were equally capable of accounting for dyadic choice and even produce the holy grail of joint decision making, the two-heads-better-than-one effect. However, they made very different predictions for joint confidence. Qualitative (see Figure 4) and quantitative (see Figure 5) comparison with the 4 strategies predictions to the empirical data showed that dyadic behavior was best described by the algebraic sum of signed wagers bounded by the maximum wager. Importantly, the same analysis showed that dyads would have earned significantly more if they followed a cognitively much simpler, less nuanced strategy of simply betting the maximum wager on every dyadic choice (irrespective the state of individual confidences). Dyad did not follow this very simple and beneficial strategy. Although maximizing earnings, dyadic wagers based on this strategy would be devoid of any metacognition and bear no information about the likelihood of correct dyadic response (Figure S12). The dyads seemed to have traded off financial gain in return for better interpersonal sharing of subjective information and matching their joint confidence to probability of correct choice. Future research would be needed to see whether this trade-off between monetary reward and richness of communication can be taken to imply that communication is of inherently value.

Interestingly, the linear independence of social and perceptual factors’ contribution to joint confidence (see Figure 3C) is also inconsistent with pure application of the bounded summing strategy. Whereas optimal cue combination would have predicted a stronger consensus effect under Null (vs. Standard) condition, the bounded Summing strategy would entail the opposite: larger change in wagering after agreement versus disagreements for Standard compared to Null trials. This prediction arises because individual are more likely to wager higher under the Standard condition (see Figure 2B, left panel). To directly compare the predictions of the bounded summing strategy for the data showing linear separability of social and perceptual factors (i.e., Figure 3C), we performed the same ANOVAs that was done for empirical data but this time for the nominal dyadic data arising from application of the bounded Summing strategy to the individual wagers (Figure S13). The results showed that if dyads were employing this strategy purely, a highly significant interaction between social and perceptual factors would be expected, *F*(1, 31) = 34.16, *p* < .001, η_G_^2^ > 0.03, in the opposite direction to that predicted by the optimal cue integration. This shows that empirical dyads are unlikely to have adopted a pure bounded Summing strategy to aggregate their judgments.

The lack of interaction in either direction could, of course, be real or a type II error. In the Null trials, the effect predicted by optimal cue combination theory may have been too weak to be observed since *both* participants did not receive perceptual evidence. Thus, even if they wanted to rely on their partners (as normative models would suggest), their partners could not offer anything but weak and unreliable evidence themselves.^1^ However, the fact that linear mixed-effects analysis—with its higher power—confirmed the same result offered some encouragement that type II errors might be unlikely. These results call for future research on confidence aggregation and using more sophisticated models than those proposed and tested here. For example, dyadic behavior might be better described by mixture of both optimal-cue combination and bounded summing. Differences between these two models must yet be better understood.

### Interindividual Differences in Metacognition and Collective Decision Making

Pervious works in collective decision making based on sharing confidence (Bahrami et al., 2010; Migdal et al., 2012) assumed that interacting agents have a good grasp on their internal uncertainty and can reliably communicate the probability that their choice is correct. Here we revisited this assumption and showed that variations in interindividual differences in human metacognitive ability (Fleming et al., 2012, 2010; Song et al., 2011) make a significant impact on collective decisions. Moreover, those previous works (Bahrami et al., 2010; Koriat, 2012; Migdal et al., 2012; Sorkin et al., 2001) invariably focused on how the collective’s choice, that is to say first-order performance sensitivity can be predicted from first-order sensitivity of the individuals making up the collective. However, previous work on metacognitive sensitivity has repeatedly shown correlations between first- and second-order sensitivity (Koriat, 2012; Kruger & Dunning, 1999; Song et al., 2011). Consequently, whether second-order metacognitive sensitivity (e.g., as measured here by type II A_ROC_) predicts success in interactive decision making was not previously known. The dual staircase paradigm we employed here served two purposes: first, it allowed us to assess individuals’ second-order, metacognitive sensitivity unconfounded by first order performance. Second, it also ensure that individuals could not arbitrate their disagreements based on the number of errors each made, leaving them only with the option to truly consult their shared metacognitive information to resolve the disagreement.

We showed that average dyadic metacognitive sensitivity did indeed predict collective benefit and performance. These results confirm that the previous assumption of uniformly similar metacognition (Bahrami et al., 2010; Koriat, 2012; Migdal et al., 2012; Sorkin et al., 2001) was too optimistic. The results are consistent with a more recent finding that investigated the dyad members’ attitude toward competence gaps between themselves and their partner (Mahmoodi et al., 2015). Interacting agents behaved *as if* they were equally competent even when ample objective evidence for the opposite conclusion was presented to them. In retrospect, it seems ironic that the theoretical assumptions made (some of them by the authors of the current paper) to understand collective decision making and the implicit bias held by the participants engaged in those studies were similar.

The use of the staircase ensured that across participants, there was no correlation between choice accuracy and metacognition. However, one may correctly argue that this relationship is still maintained *within* each participant. A given participant is more likely to be right in trials he wagered high versus low. Having to go through a staircase would not break down the trial-by-trial relationship between confidence and accuracy. We completely concur with this notion. In fact, metacognitive sensitivity as measured here is an attempt to capture that trial-by-trial association. Furthermore, we take this idea one step further to suggest that the trial-by-trial association between accuracy and confidence is at the heart of the two-heads-better-than-one effect, which thus depends on metacognition. The linear mixed-effects analysis showed that the individuals who turned out to be more influential for the final dyadic choice on each trial were also those who wagered higher, irrespectively of their first-order accuracy. People do not have *direct* access to their partner’s internal uncertainty but only to the *reported one* (confidence or wagers). Because wager judgments tracked the trial-by-trial variability in first order accuracy, dyads were able to recognize the individual with the highest chances of being correct on a given trial by following the choice with highest wager. This would yield perfect results if wager was perfectly correlated with accuracy. However, people vary in their ability to track their probability of being correct. Thus the strategy of following the highest wager would backfire if the association between confidence and accuracy is weak, that is, in participants with low metacognitive sensitivity. This is exactly what our results show: average metacognitive sensitivity of dyads was correlated with collective benefit.

### Is Collective Benefit a Purely Statistical Artifact?

It is possible that the collective benefit accrued by our dyads here is an entirely statistical artifact (Mannes et al., 2014). Our findings could in principle be attributable not to any social interaction per se but to the fact that for each dyadic decision, participants received an extra piece of independent information (i.e., partner’s opinion) whose structure of error (noise) was uncorrelated with their own estimate. Putting together samples drawn from uncorrelated noisy distributions improves one’s estimate of the true value of a random variable by averaging out the noise as long as the independent estimates bracket the true value (Armstrong, 2001; Surowiecki, 2004; Yaniv & Choshen-Hillel, 2012). However, there is ample evidence against the simple statistical impact of multiple sampling as a sufficient explanation of collective benefit in interactive joint decision-making. For instance (Bahrami et al., 2010) Experiment 4 and (Bahrami et al., 2012a) Experiment 3 found that dyads can outperform individuals only if communication is allowed (A. A. Brennan & Enns, 2013). If participants do not communicate their confidence estimates or if such communication happens without verbal interaction, then receiving an extra decision (sample) from a partner will not be sufficient for robust collective benefit to emerge. The correlation demonstrated in Figure 6 indicates that putting together the independent choices decoupled from their respective wagers would wipe out collective benefit. Thus, our results converge with previous evidence to argue that a purely statistical superposition of samples could lead to the sort of collective benefit demonstrated here.

Several measures were taken to ensure that our results were not affected by possible confounding impacts of monetary wagering (see Supplementary material). Separate measurements were taken to assess loss (De Martino, Camerer, & Adolphs, 2010) and risk aversion (Holt & Laury, 2002) in each participant to test whether these two biases affected the wagering behavior. No correlations were found between participants’ risk- and loss-aversion and individual and group measures of interest (including performance, threshold, metacognitive sensitivity and earnings) showing that these biases were unlikely to have influenced our experiment.

### Conclusions

We disentangled the effects of sensory evidence and social information on confidence formation as measured by postdecisional wagering. Social information has no perceptual value per se but offers a useful and computationally inexpensive heuristic. We showed that positive (agreement) and negative (disagreement) social information affected wager size in opposite directions and these two effects were correlated with proportional changes in joint accuracy. We also showed that collective benefit in a dyad was related to second-order ability of the participants, even though variability in first order sensitivity was kept constant. Thus a bidirectional effect was shown where social interaction modulated wagering and individual metacognitive sensitivity predicted collective success. A bounded Summing strategy reliably, although not perfectly, predicted empirical opinions aggregation. These results point out that metacognitive abilities like confidence calibration play an important role in human cooperation and interaction.

## Supplementary Material

10.1037/xge0000180.supp

## Footnotes

1 In retrospect, this issue could have been addressed if we had another condition with one participant receiving the Null and the other receiving the Standard stimulus. Such condition would be comparable with cases of multisensory capture (Alais & Burr, 2004) where the more reliable source of information completely takes over the less reliable.
