# Supplementary material for: The Perceptual and Social Components of Metacognition
Source: J Exp Psychol Gen. 2016 Jun 16;145(8):949–65. doi: 10.1037/xge0000180 (PMC4961070; doi:10.1037/xge0000180)

**Supplemental Materials**

**The Perceptual and Social Components of Metacognition**

**by N. Pescetelli et al., 2016, *JEP: General***

**http://dx.doi.org/10.1037/xge0000180**

**1. Control measures**

### In order to control for possible economic biases, measures of risk aversion and loss aversion were taken.

### *1.1. Risk aversion*

A computer-based version of the Holt and Laury’s task (Holt & Laury, 2002) was performed by both participants. In this test the subject has to indicate which lottery in a sequence of lottery pairs he would prefer to bet on. Two possible rewarding outcomes are allowed in each lottery. One lottery is “safe”, meaning that it is characterized by wins of medium size (£2 and £1.60); the other is “risky”, meaning that it is possible to win either the highest (£3.85) or the lowest (£0.10) amount of money within the game. The probability of winning one prize or the other in each lottery is gradually shifted, in order to find the point where preferences are reverted between lotteries. Usually participants are risk averse meaning that they switch from the safe lottery to the risky one only when the payoff difference is large enough, so that there is only an extremely scarce chance of getting the smallest reward. In our version of the task, the test was presented on screen, with lottery pairs disposed in a randomized order three times each in order to check for response consistency. A pie chart for each lottery was also displayed to make the task intuitive (Figure S1).

### *1.2. Loss aversion*

A test for loss aversion assessment was also given to participants. Loss aversion generally refers to the different emotional impact that gains and losses have on people (Kahneman & Tversky, 1979). The pleasure generated by a win is usually smaller than the regret generated by a loss of the same magnitude. In order to assess loss aversion we used the coin-flip gamble task used by (De Martino et al., 2010). In this task a matrix of gambles with different expected values is presented to the participant. Each gamble is a coin toss with 50% chance of winning or losing money. Although the probability of loss and win are the same, the amount of money that subjects can gain or lose is varied in each gamble. The subject’s task is to accept or refuse to flip the coin (Figure S2). At the end of the test only one among the accepted gambles is selected by the computer and the relative amount is added or subtracted from the subject’s credit. Although expected values are positive every time the win-side is higher than the loss-side, people are usually loss averse, meaning that they refuse to gamble unless the potential win is far greater than the potential loss (Sokol-Hessner et al., 2009). In our version of the task the range of wins/losses was between £2.20 and £5 in steps of £0.20. Such choice was motivated by the fact that we wanted to make magnitudes in this task and in the PDW task comparable.

### *1.3. Questionnaires*

After completing two runs of trials, each participant completed the Empathy Quotient questionnaire (Baron-Cohen & Wheelwright, 2004). This questionnaire assesses people’s focus on social relations and attention to social cues.

A second questionnaire, the BIS/BAS test (Carver & White, 1994), was sent by e-mail to participants. The BIS/BAS questionnaire assesses activation and inhibition personality traits; sub-scales have been linked with perception of social threats, dominance and gaze-avoidance (Putman, Hermans, & van Honk, 2004; Stewart et al., 2012; Terburg, Hooiveld, Aarts, Kenemans, & van Honk, 2011).

### 2. Results

### *2.1. Risk Aversion*

A RA-index was computed for each subject. This was obtained by regressing participant’s answers in the Holt & Laury task using a cumulative normal distribution. The MATLAB function glmfit(link=probit) was used. RA-index was defined as the point of subjective equality (PSE = 50%), i.e. the point when choosing Lottery-A or Lottery-B was indifferent for the subject. The average value across subjects was *M* = 5.88; *SD*= 1.8691.

Individual values were compared against individual and group variables of interest to look for possible effects. No correlation was found significant.

### *2.2. Loss Aversion*

Similarly a LA-index was computed. A logistic regression (De Martino et al., 2010) of subjects’ responses using The MATLAB function glmfit(link=logit) was used. Again, PSE was used as loss aversion measure, representing absolute expected values of gains over losses. The average value was *M* = 0.23; *SD* = 0.36. This means that on average subjects accepted all gambles where the gain was at least 23p superior to the loss.

Individual values were compared against individual and group variables of interest to look for possible effects. No correlation was found significant.

### *2.3. Risk Aversion*

Empathy questionnaires were scored as in literature (Baron-Cohen & Wheelwright, 2004). The average results are in line with scores reported for male population (ibid.): *M* = 39.41, *SD* = 11.22. Again, all correlations with individual and group measures were not significant suggesting that empathy was not a relevant variable in our paradigm.

The BIS/BAS questionnaire was scored as indicated at <http://www.psy.miami.edu/faculty/ccarver/sclBISBAS.html>. Two dyads (Dyad 11 and Dyad 24) did not reply to the form that was sent, so were excluded from the analyses. BIS subscale was negatively correlated with individual accuracy (*r*(30) = -.402; *p* = .03). BIS subscale detects anxiety traits in situations where the person has to face a social conflict or adverse circumstances. Interestingly inhibition-anxiety traits have been linked with pre-attentive/attentional bias for highly aversive stimuli and avoidance of mildly-aversive stimuli (Mogg & Bradley, 1998) suggesting that facing a social situation of conflict might impose a cognitive burden on subjects’ performance.

### 3. Multilevel analysis of social and perceptual effects on wager size

Both our ANOVA analysis (Figure 3C) and multilevel regression on dyadic wager size showed a linearly additive impact of perceptual and social information on dyadic wager. However, results from these two analyses were included different sets of trials. In particular, the multilevel regression reported in the main text (p.25) includes Conflict trials, which are not used for the ANOVA analysis. It might be that the result found at the trial-by-trial level is due to the inclusion of Conflict trials that capture some of the variance of the dependent variable. We thus replicated the main model (Table 1a) after removing Conflict trials. The model included dyadic wager size as dependent variable and several independent predictors: consensus (categorical, 1 = agreement, 0 = disagreement), stimulus presence (categorical, Standard vs Null condition), individual wager and their reciprocal interactions as fixed-effects. Trials were grouped within individuals who in turn were grouped within dyads. Consensus, individual wagers, stimulus presence and the interactions of consensus with the other two predictors were defined as random effects and thus were allowed to vary for each participant. Only a random intercept was defined for dyads. The results show that greater dyadic wager sizes were strongly predicted by consensus (*β* = 1.32, SE = 0.18, *β*_std_ = 0.99, SE_std_ = 0.06, *p* < .001), stimulus presence (*β* = 0.57, SE = 0.10, *β*_std_ = 0.22, SE_std_ = 0.05, *p* < .001), and individual wager size (*β* = 0.40, SE = 0.04, *β*_std_ = 0.36, SE_std_ = 0.03, *p* < .001). Critically, there was no interaction between stimulus presence and consensus (*β* = -0.008, SE = 0.09, *β*_std_ = -0.005, SE_std_ = 0.06, *p* = .93), thus replicating the ANOVA results (Figure 3C) and confirming the linearly additive effects of social and perceptual components of confidence. A negative interaction was observed between stimulus presence and individual wager size (*β* = -0.07, SE = 0.02, *β*_std_ = -0.06, SE_std_ = 0.02, *p* = .001). It suggested that the effect of presence of stimulus made smaller contribution when individual wager size was large. Individual wager size positively interacted with consensus (*β* = 0.11, SE = 0.05, *β*_std_ = 0.10, SE_std_ = 0.05, *p* = .04) suggesting again that individual wagers better predicted dyadic wagers under agreement than disagreement (c.f. Opinion Space in empirical and nominal dyads).

**Supplementary Material Figures**

**FIGURE S1**

Holt & Laury task for risk aversion. Subjects have to choose between two rewarding lotteries with different payoff. Across trials the amount of wins remains constant but the outcome probabilities change.

**FIGURE S2**

Loss aversion task. Subjects have to accept or refuse to gamble on a coin toss, where one side represents a win and the other side represents a loss.

**Figure S3**

Individual wager distributions in Standard trials. Subjects 1 and 2 belong to Dyad1, subjects 3 and 4 to Dyad2 and so on.


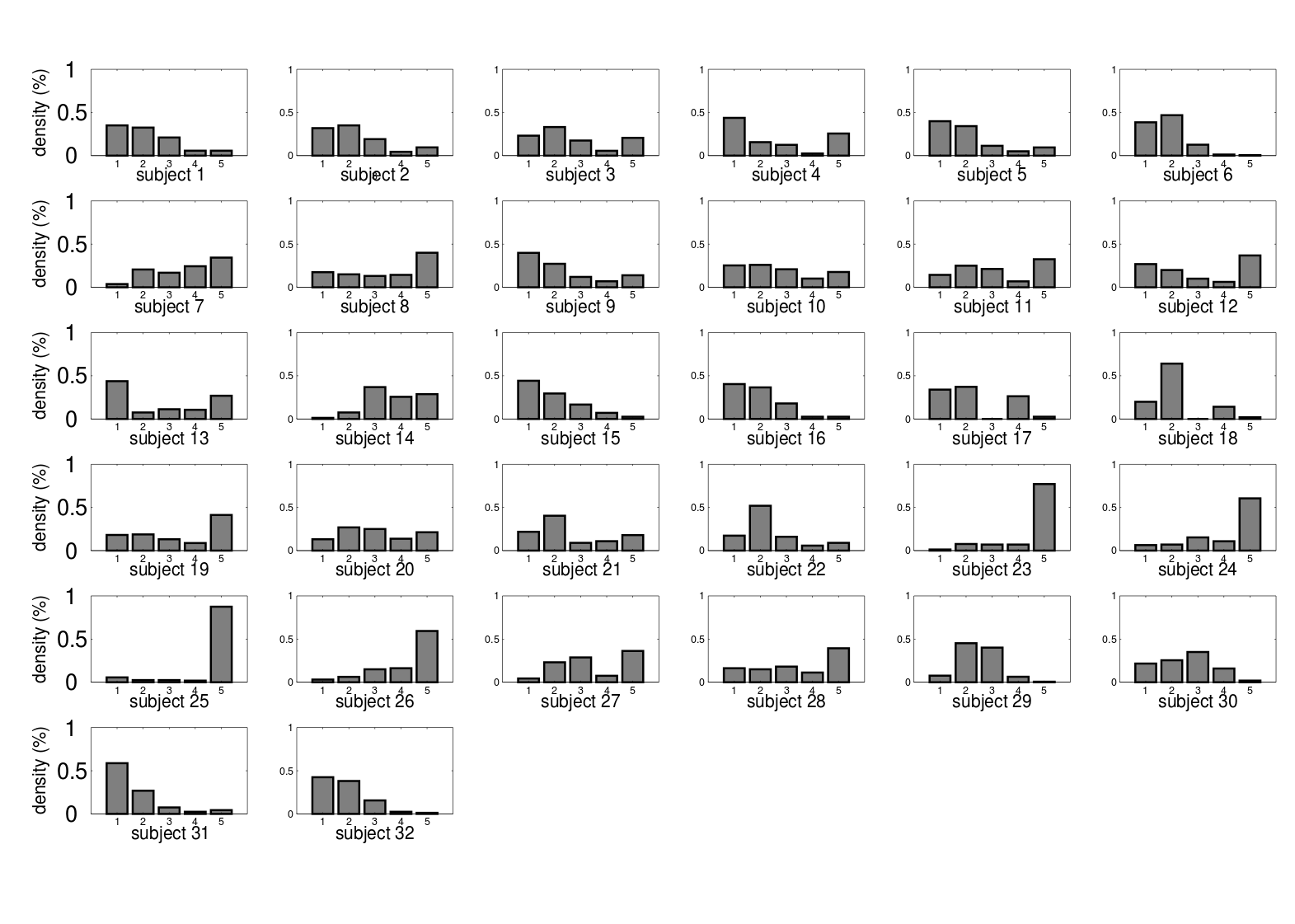


**Figure S4**

Dyadic wager distributions in Standard trials.


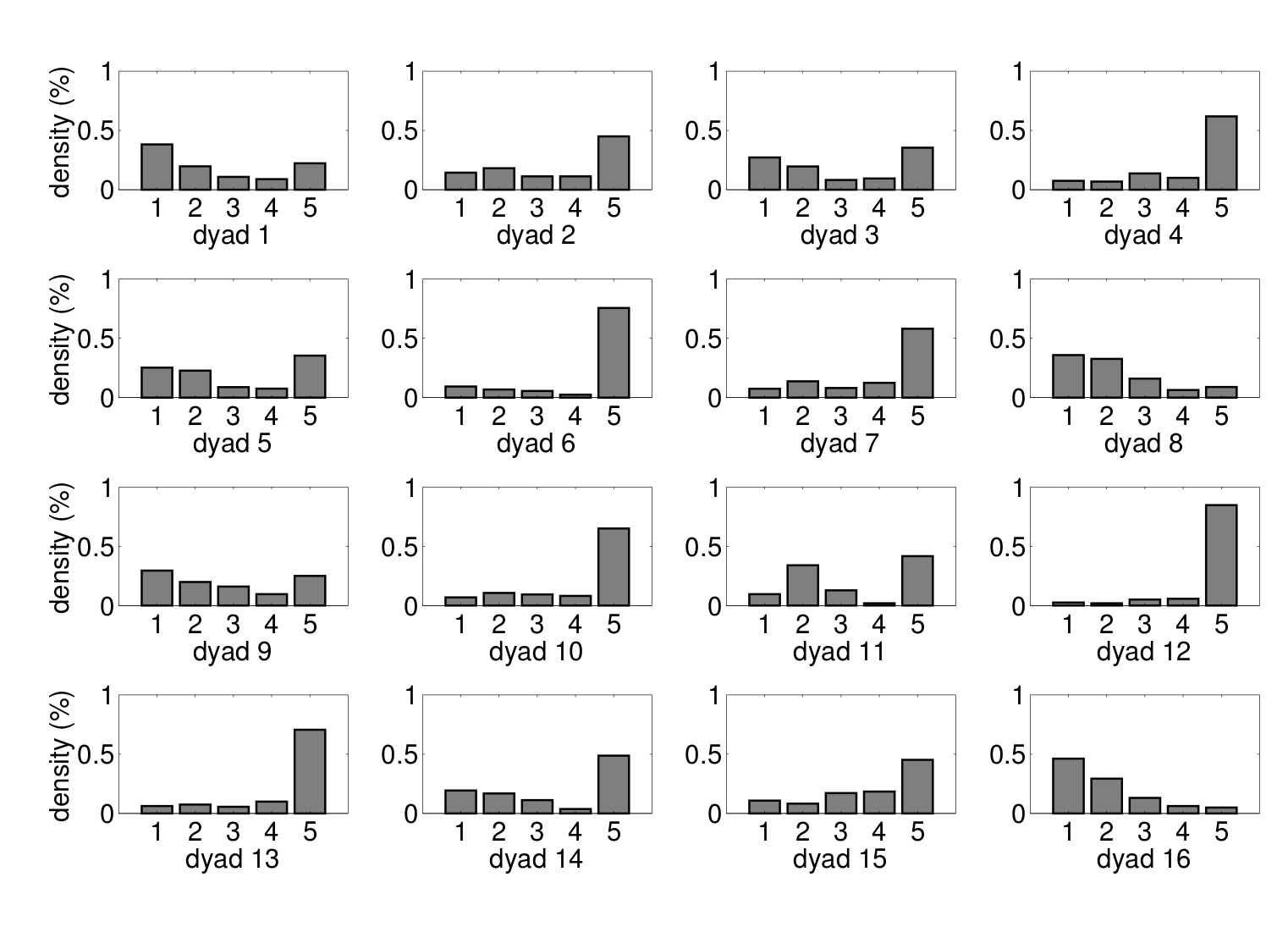


**Figure S5**

Individual wager distributions in Conflict trials. Subjects 1 and 2 belong to Dyad1, subjects 3 and 4 to Dyad2 and so on.


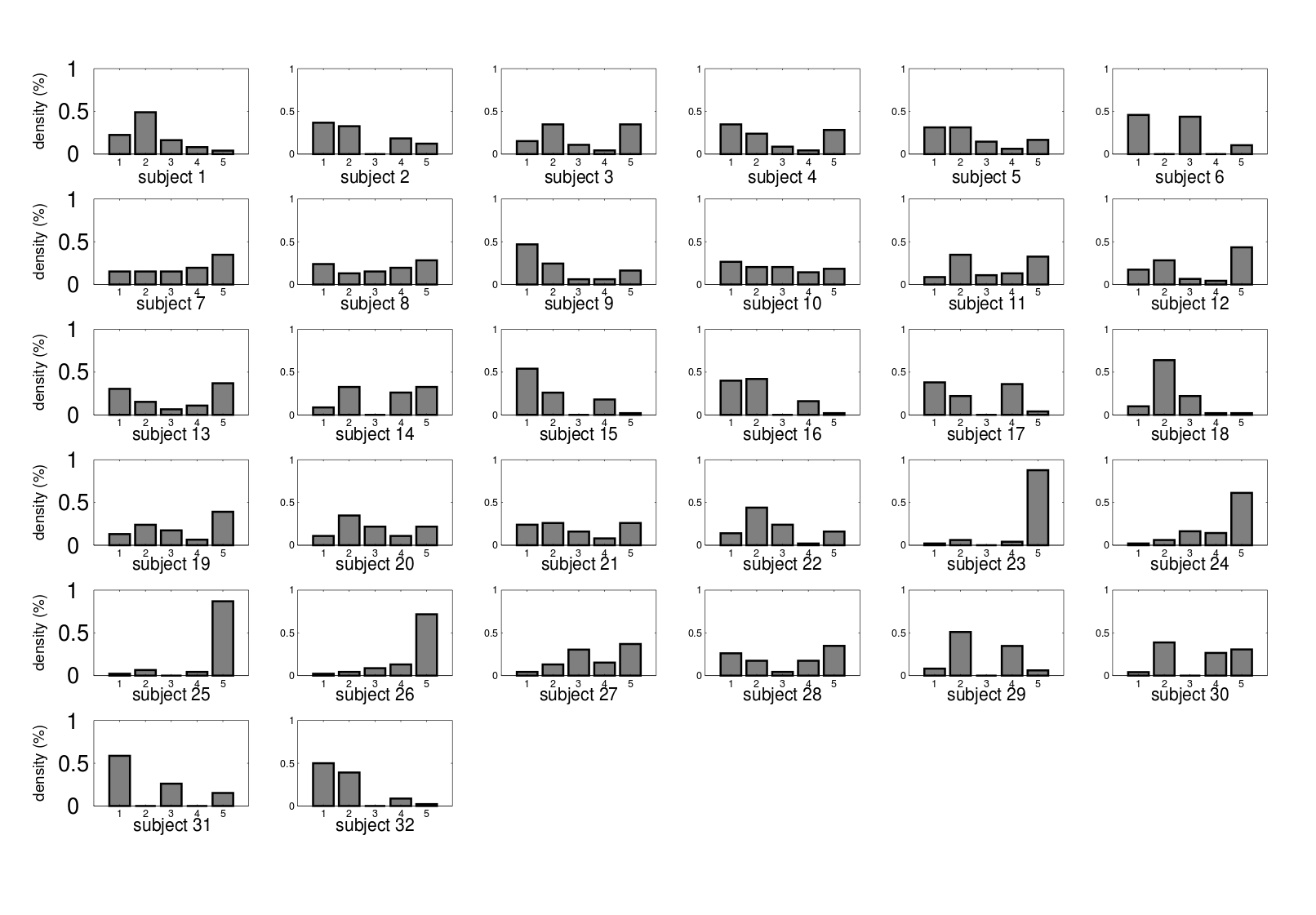


**Figure S6**

Dyadic wager distributions in Conflict trials.


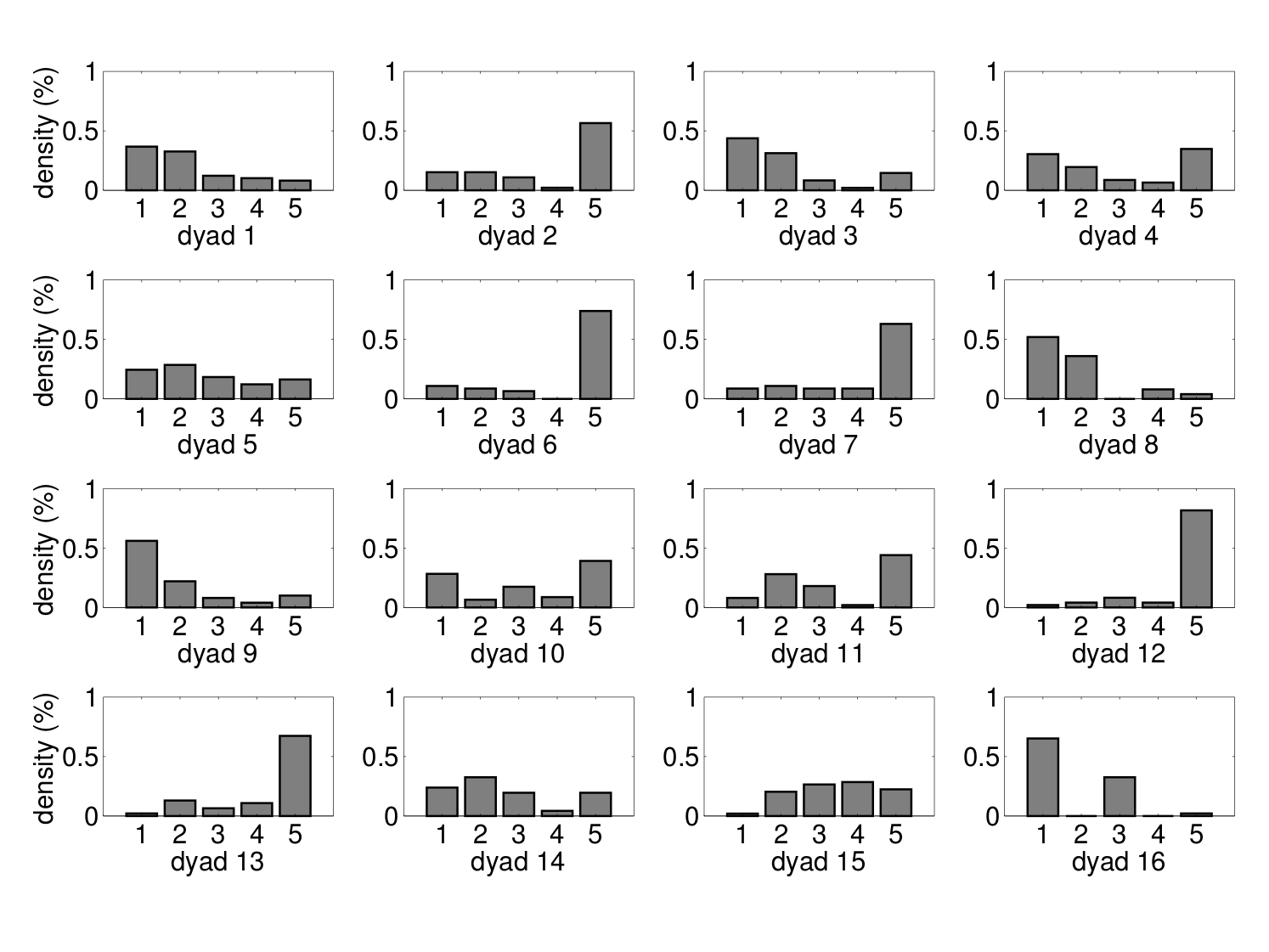


**Figure S7**

Individual wager distributions in Null trials. Subjects 1 and 2 belong to Dyad1, subjects 3 and 4 to Dyad2 and so on.


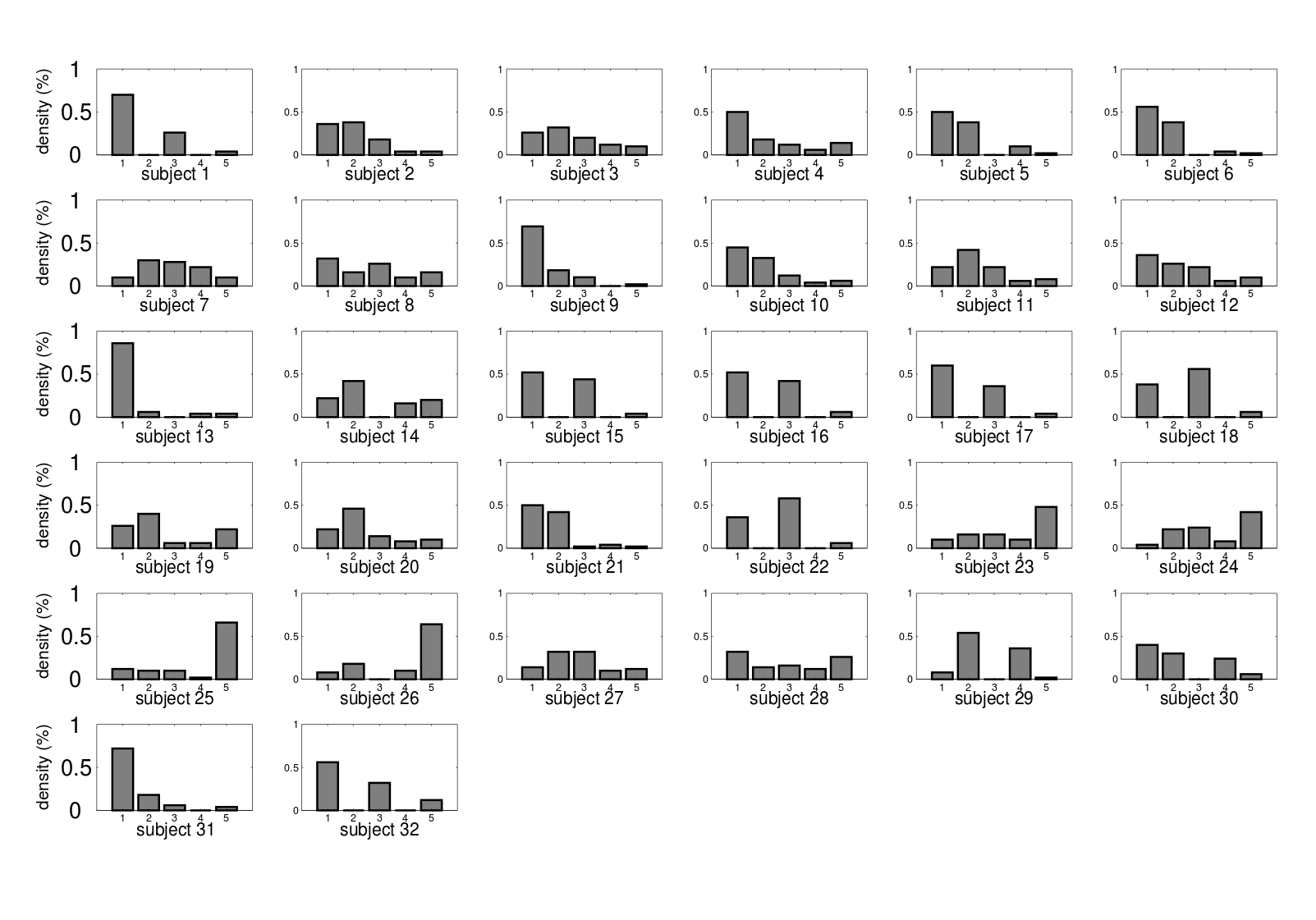


**Figure S8**

Dyadic wager distributions in Null trials for each dyad.


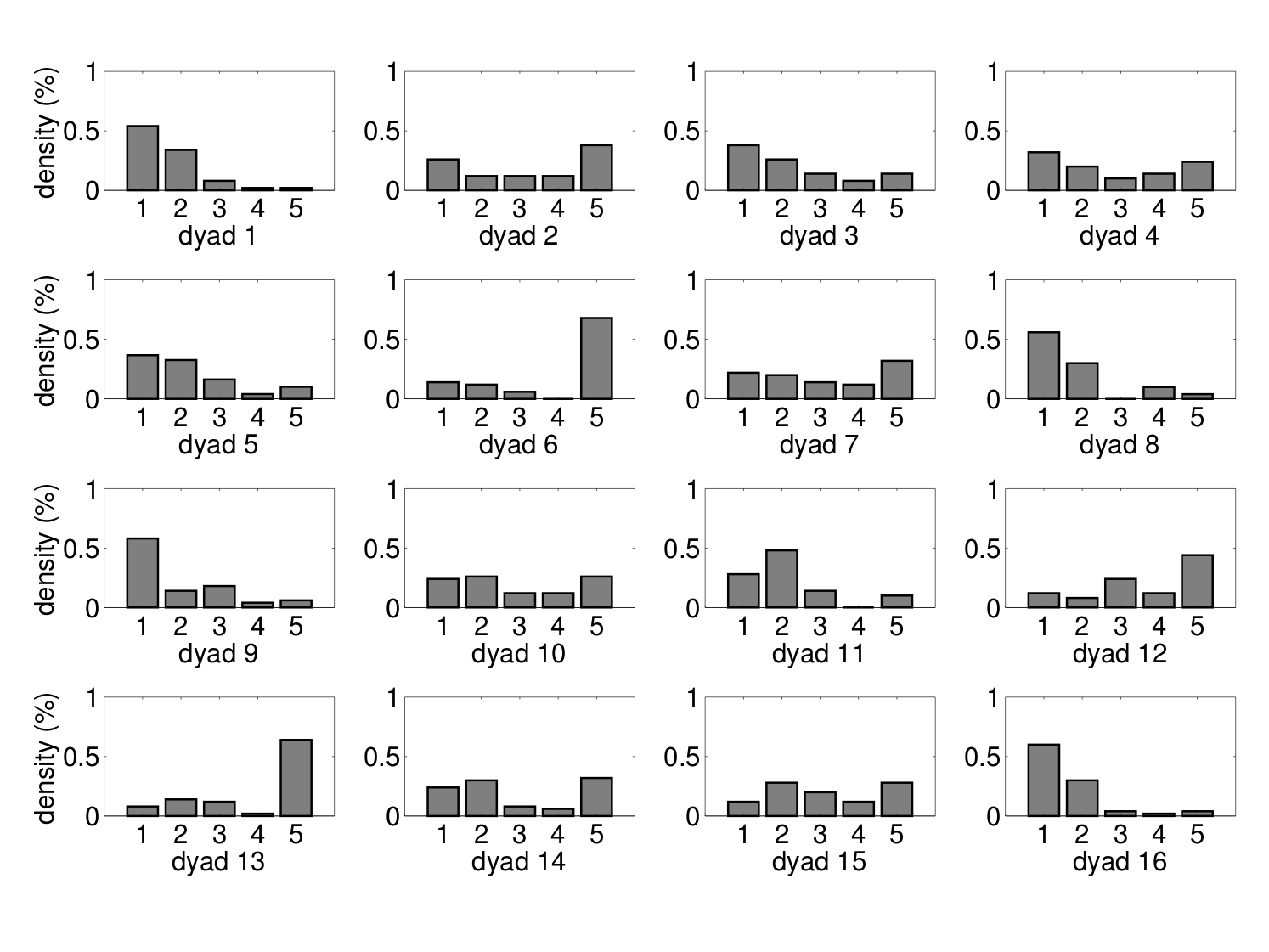


**Figure S9**

**A)** Individual accuracy and metacognitive sensitivity grouped by dyad. This figure is the same as Figure 6A but ordered by dyad instead of by ascending order in individual metacognition. **B)** This figure is the same as in Figure 6B but with individual metacognitive sensitivity measures superimposed on average metacognitive sensitivity within each dyad.


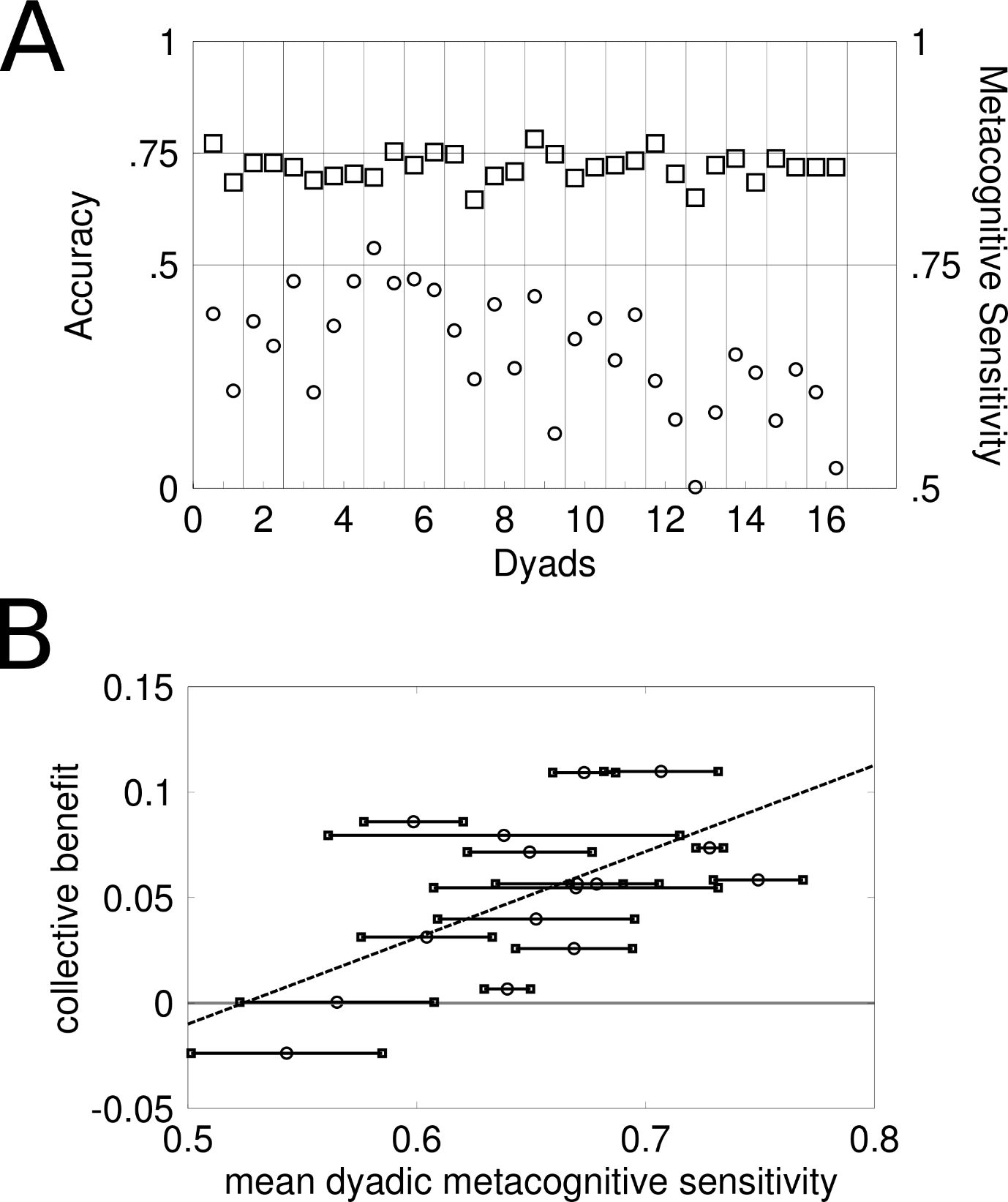


**Figure S10**

Opinion Space for COINFLIP strategy. This algorithm selects on each trial a participant’s interval and wager size at random. Notice how this is a noisy version of the Averaging strategy.


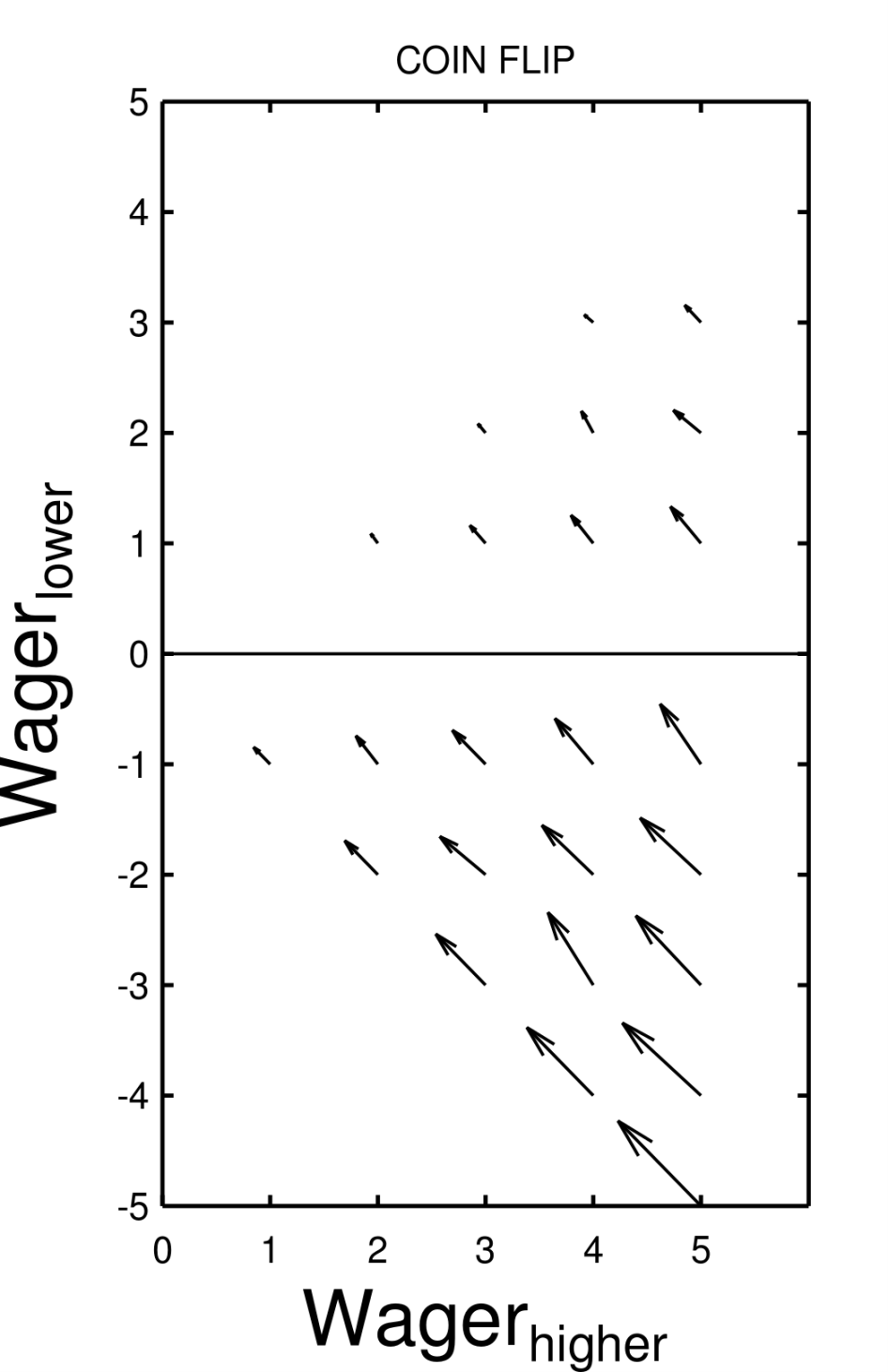


**Figure S11**

Opinion Space for each dyad. Given the symmetry of the data, results from the two intervals have been collapsed together. X-axis represents the private wager of the more confident individual on each given trial; y-axis represents the private wager of the other individual, relatively to the first individual’s wager on a given trial. The upper part represents situations when the two participants agree on the same interval, while the bottom part represents situations of disagreement. The 16 plots show the Opinion Space for each dyad. It can be noticed that all dyads seem to adopt a Summing-like opinion aggregation strategy, although data are noisier (e.g. missing cells).


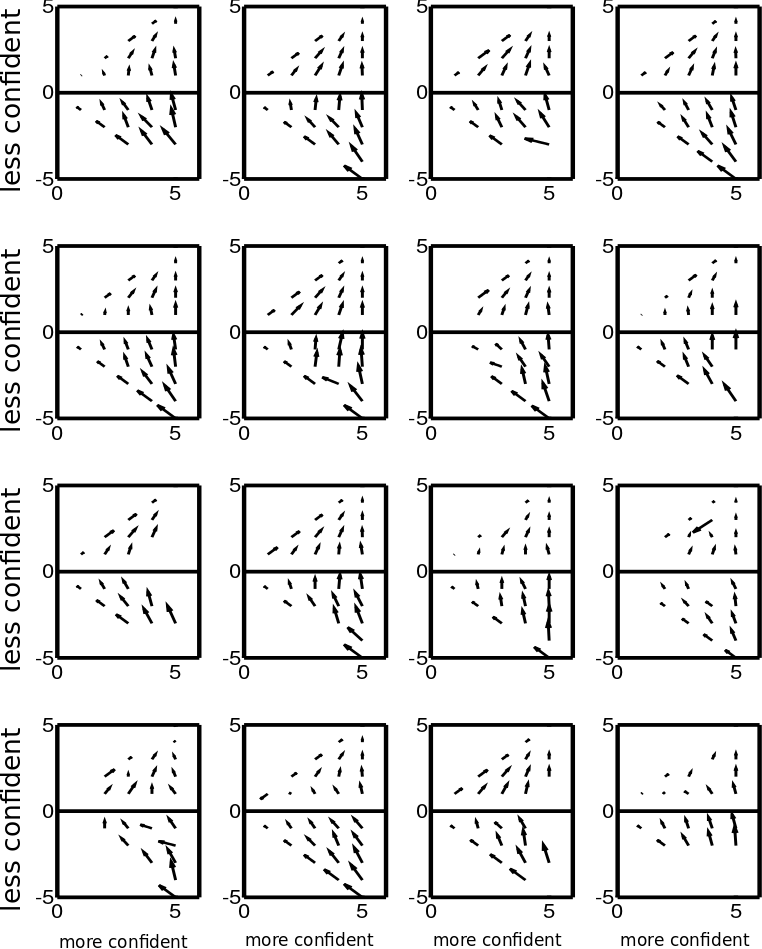

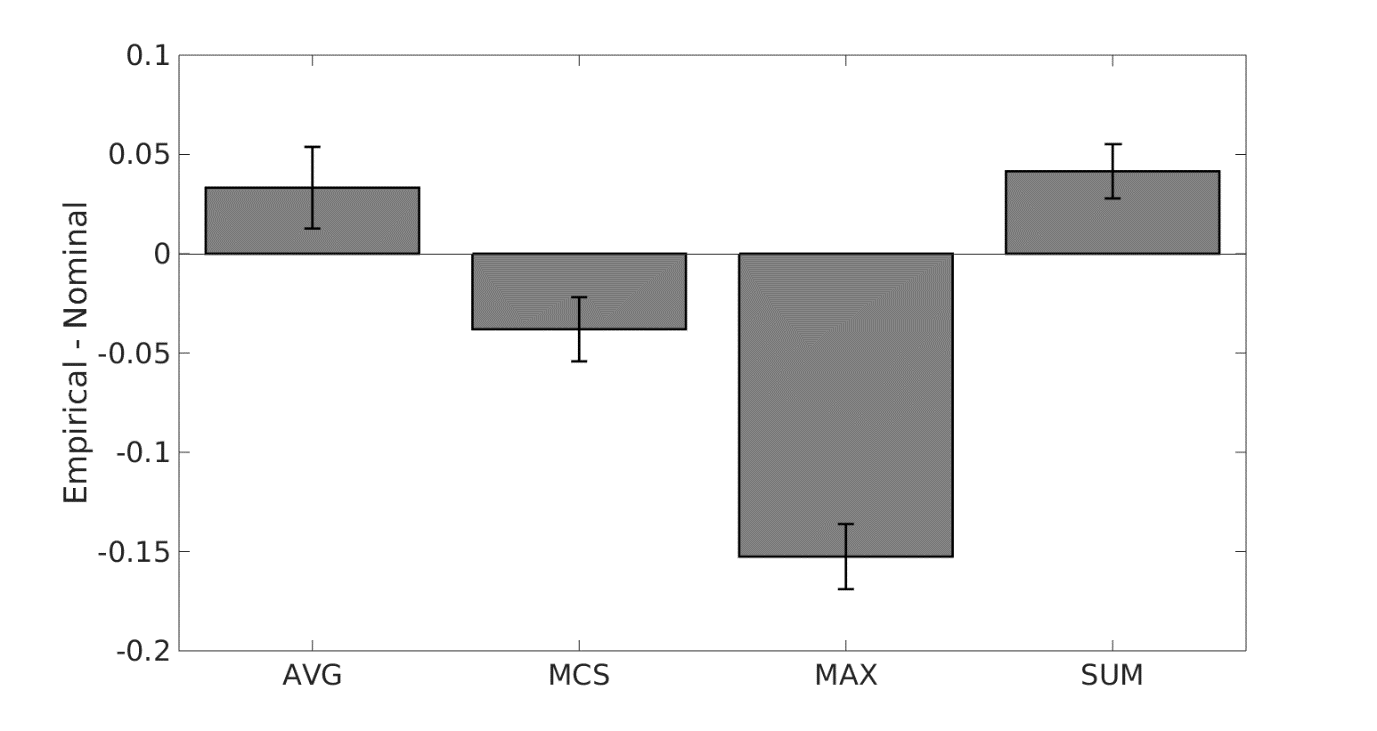


**Figure S12.**

**A)** Metacognitive sensitivity - as measured by type II AUROC- between empirical and nominal. The plot shows how MCS and Maximize strategies underperformed in terms of second-order accuracy compared to empirical dyads, while Average and bounded Sum slightly over-performed.

**Figure S13.**

**A)** Social versus perceptual effect on wager size for a purely Summing strategy. Together with the already reported social and perceptual effect, now also the interaction between the two is significant (*F*(1,31) = 34.16, *p* < .001, *η_G_^2^* > 0.03). The two conditions differ in agreement (*t*(31) = 8.37, *p* < .001) but not in disagreement (p > .1)

**B)** Social versus perceptual effect on wager change for a purely Summing strategy. The interaction between social and perceptual effect is significant (*F*(1,31) = 28.32, *p* < .001) as well as the social effect (*F*(1,31) = 1e3, *p* <.001) and the perceptual effect (*F*(1,31) = 8.16, *p* < .01)


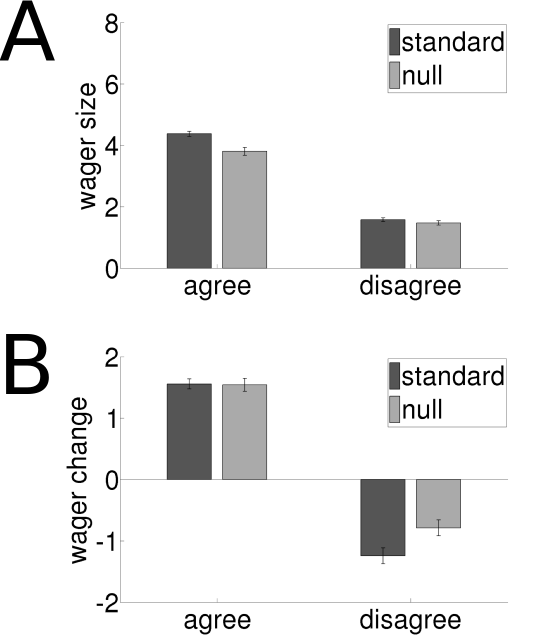


**Figure S14.**

**A)** Wager change from pre to post interaction in agreement and disagreement for the three conditions. The distance between agreement and disagreement within each condition is the consensus effect. Notice how Standard and Null conditions do not differ (although there is a numerical difference for agreement only) while wager change is significantly lower for Conflict agreement.


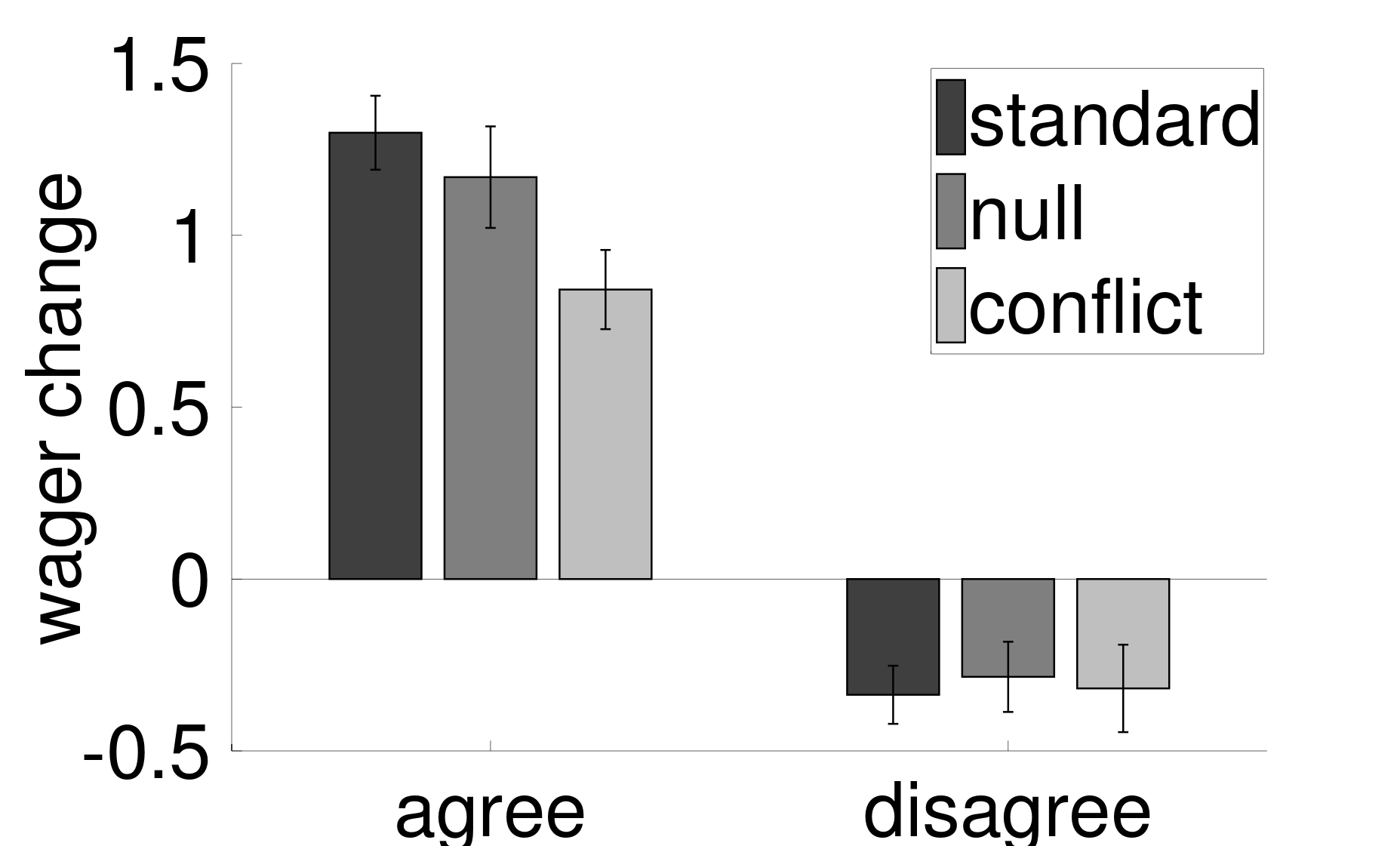


**Multilevel Linear Mixed-Effects Models**

**Table S1a**

Multilevel Linear Mixed-Effects Model on dyadic wager size. All conditions included. 0 = Null, 1 = Standard, 2 = Condition.

Formula:

abs_dyadic_wager = 1 + consensus*condition + indiv_wager* consensus + indiv_wager*condition + (1|dyad) + (1 + consensus *condition + consensus *indiv_wager | subject:dyad)

FitMethod: REML

Notice that the notation with the “*” symbol represents all the combinations of factors on the two sides of the symbol “*”. For instance: y ~ 1 + consensus *condition is equivalent to the more verbose formula y~ 1 + consensus + condition + consensus:condition, where consensus:condition represents the interaction between these two terms.
The upper table has been computed with unstandardized data, while the lower one with standardized one.


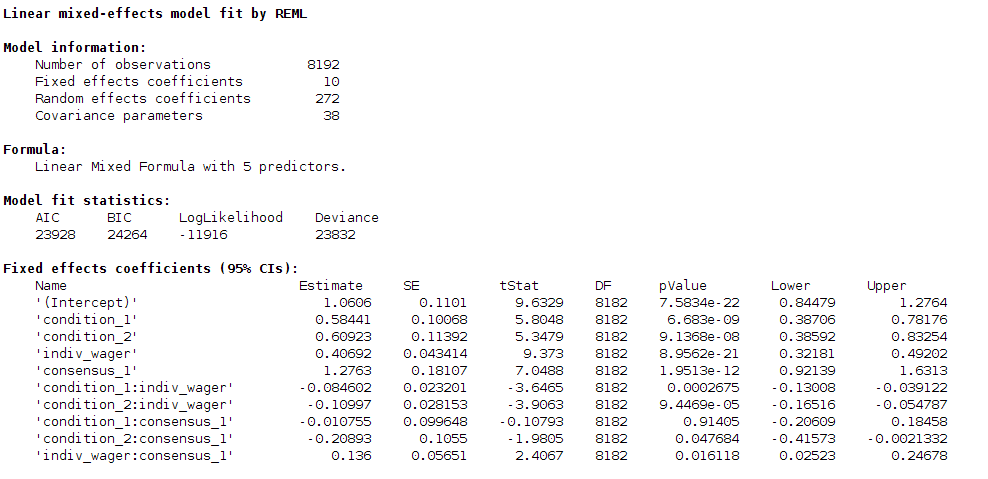

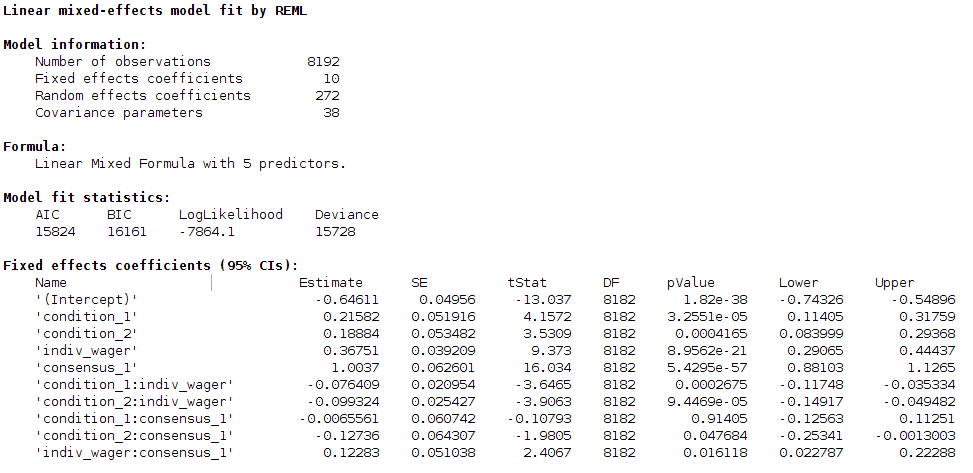


**Table S1b**

Same Multilevel Linear Mixed-Effects model as in Tables S1a but with the addition of deliberation time (RT), defined as the time passed between the last individual wager and the dyadic wager.

abs_dyadic_wager = 1 + consensus*condition + indiv_wager*consensus+ indiv_wager*condition + RT*indiv_wager +(1|dyad) + (1 + consensus*condition + consensus*indiv_wager | subject:dyad)

FitMethod: REML

The upper table is computed with unstandardized data, lower table with standardized one.


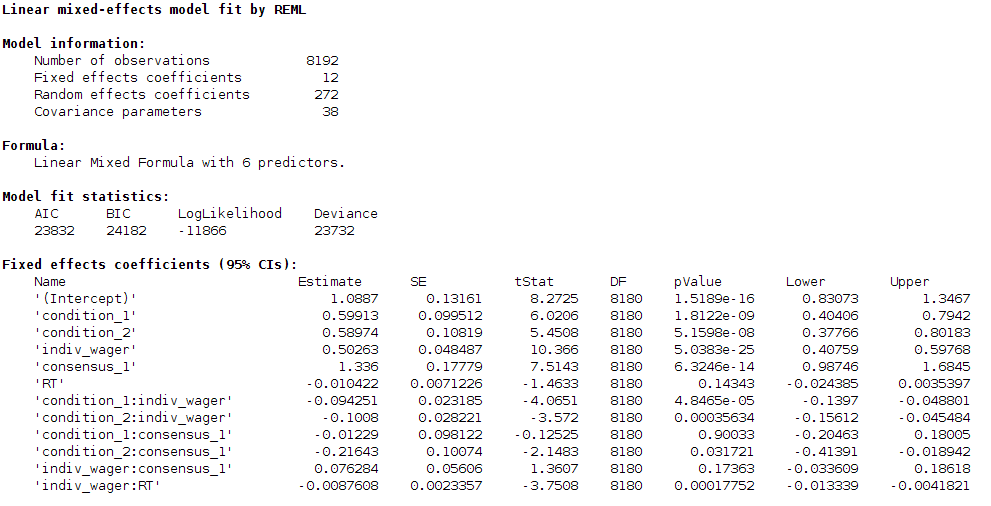

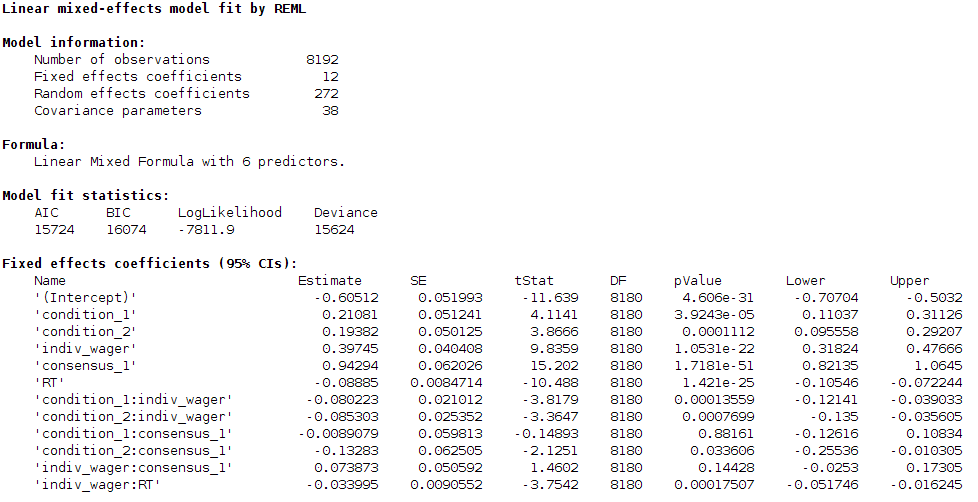

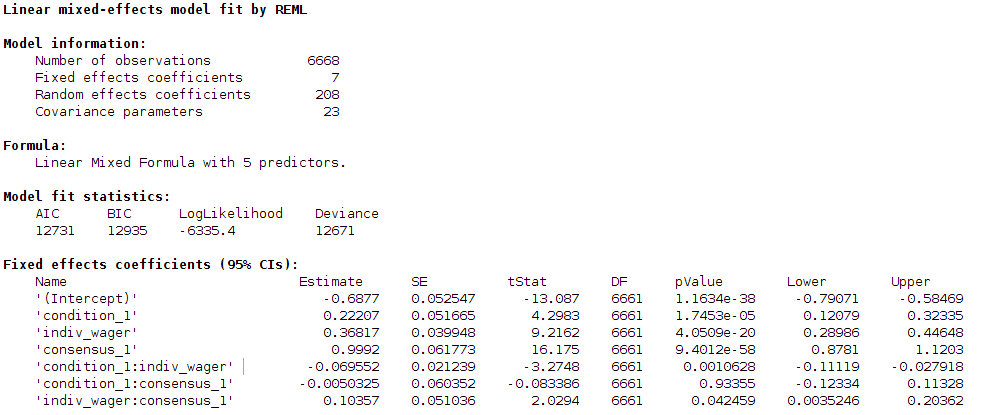


**Table S2**

Perceptual vs social components of post-decisional wagering. Same coding as in the previous tables.

Formula:
abs_dyadic_wager = 1 + consensus*condition + indiv_wager* consensus + indiv_wager*condition + (1|dyad) + (1 + consensus *condition + consensus *indiv_wager|subject:dyad)

Fit method: REML

The upper table is computed with unstandardized data, lower table with standardized one.


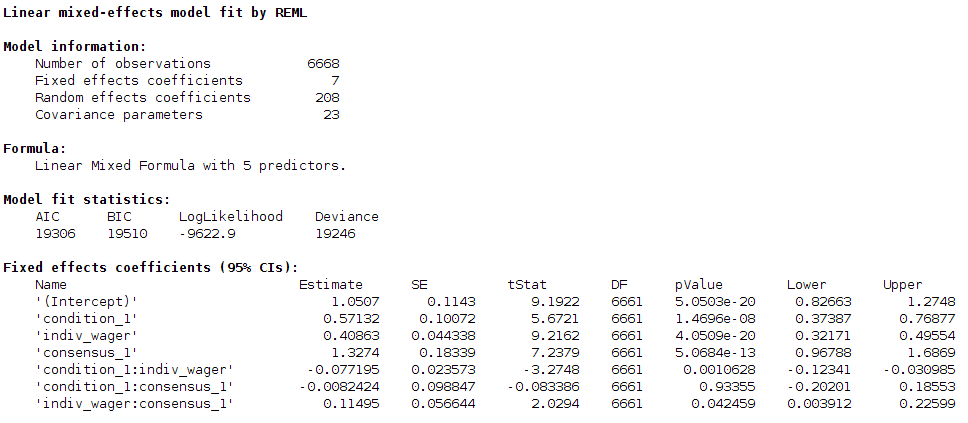

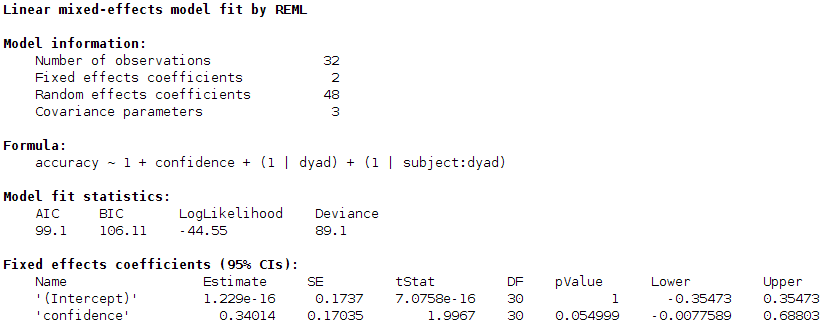

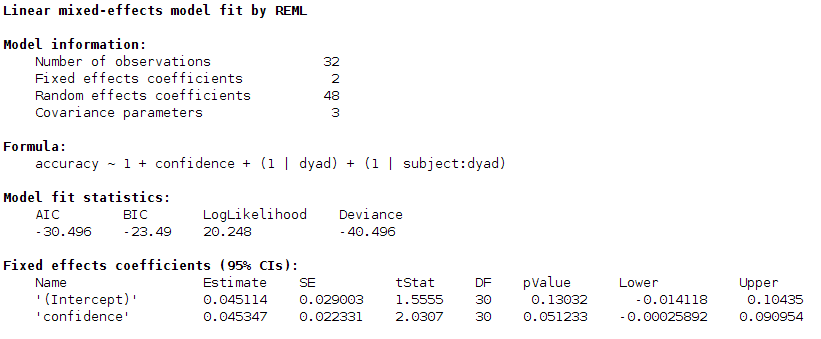


**Table S3**

Here we test whether the difference between promise of consensus and warning of disagreement in correct response rates can be predicted by the difference between promise of consensus and warning of disagreement in wager sizes. *Accuracy* refers to the difference between promise of consensus and warning of disagreement for accuracy rates. *Confidence* refers to the same difference but for post-decisional wagers. The model wants to be a stricter check for the correlational analysis reported in the main text.

Formula :

accuracy = 1 + confidence + (1|dyad) + (1|subject:dyad),

Fit method: REML

Upper table is computed from unstandardized data, lower table is computed from standardized one.

**Table S4a**

Formula:

influence = 1 + condition*indiv_wager + condition*earnings + (1 | dyad) + (1 | subject:dyad)

Fit method: REML. Conditions are coded as 0 = Null (reference), 1 = Standard, 2 = Conflict.

Upper table is computed from unstandardized data, lower table is computed from standardized one.


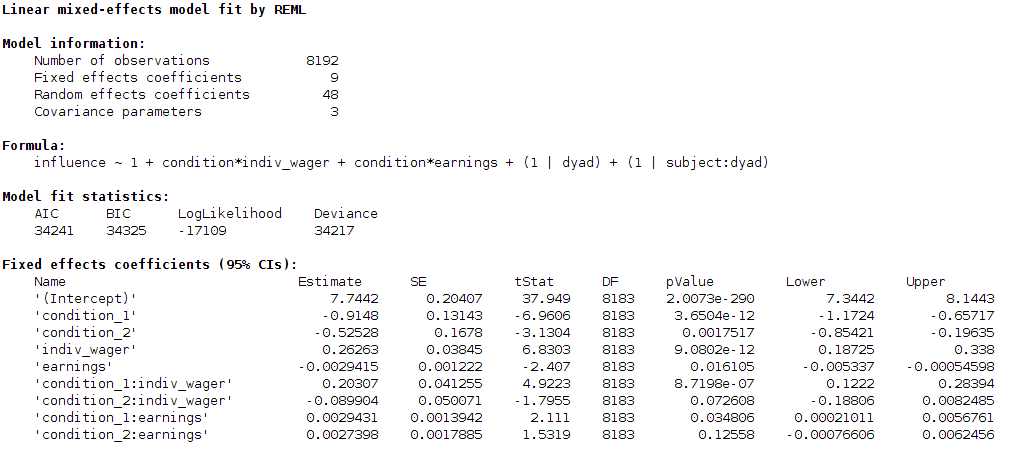

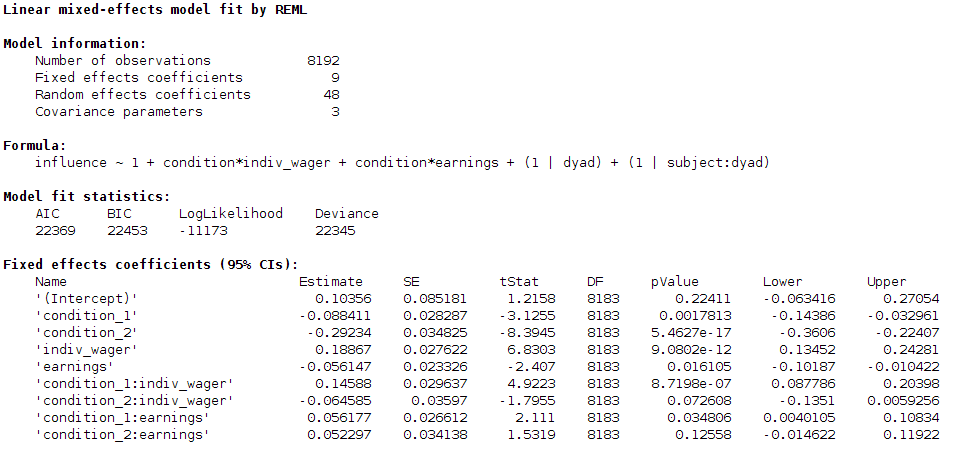

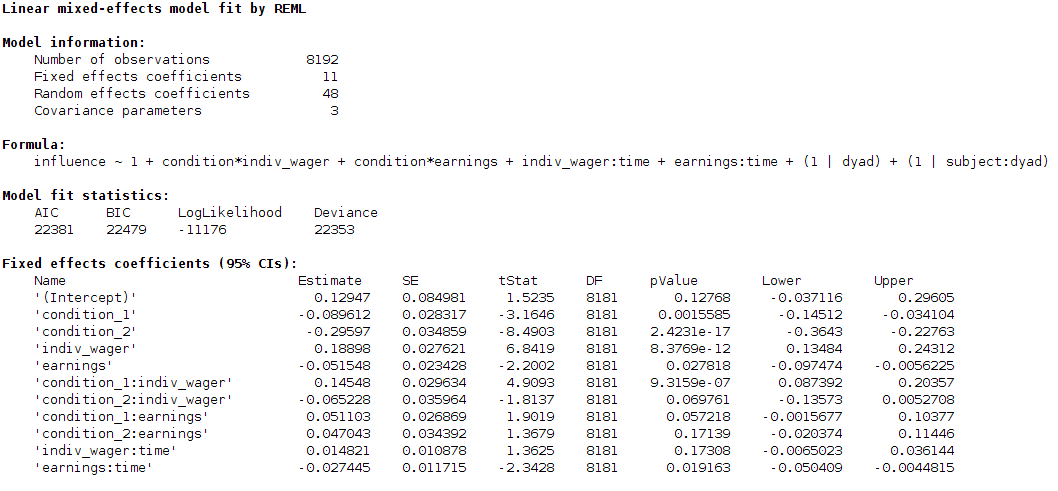


**Table S4b**

The addition of time among the predictors shows that participants fail to learn who is the more metacognitively accurate person in the dyad even if feedback was provided for most of the trials. On the contrary the relation between metacognitive sensitivity and influence becomes smaller over time as shown by the negative coefficient for the earnings*time interaction term.

Formula shown in the tables. Upper table was computed from unstandardized data, lower table was computed from standardized one.


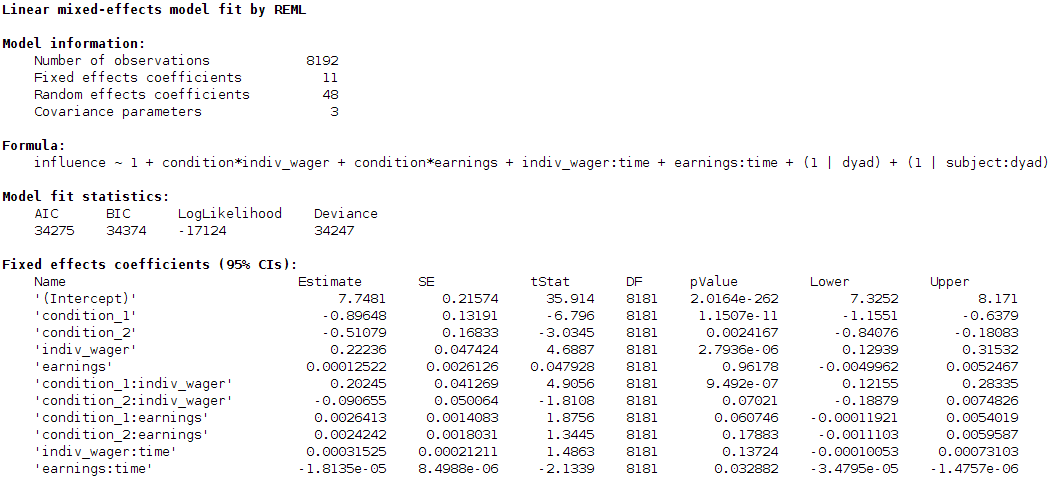

Supplement: Supplementary file 1 [file zfr005162771sb01.docx]
